# m^5^C Reader ALYREF Promotes the Progression of Endometrial Cancer by Activating the XRCC6‐Mediated Wnt/β‐catenin Signaling Pathway

**DOI:** 10.1002/advs.77548

**Published:** 2026-09-27

**Authors:** Linlin Hao, Jian Zhang, Qi Qian, Min Jiang, Yu Liu, Yan Geng, Nan Cui, Qi Guo, Jie Guo

**Affiliations:** ^1^ Department of Radiation Oncology The Second Hospital of Jilin University Changchun China; ^2^ Department of Radiation Oncology The Affiliated Wuxi People's Hospital of Nanjing Medical University Wuxi Jiangsu China; ^3^ Wuxi Medical Center Nanjing Medical University Wuxi Jiangsu China; ^4^ Wuxi People's Hospital Wuxi Jiangsu China; ^5^ School of Life Sciences Department of Biology Southern University of Science and Technology Shenzhen China

**Keywords:** ALYREF, endometrial cancer, m^5^C, Wnt/β‐catenin pathway, XRCC6

## Abstract

5‐Methylcytosine (m^5^C) is a vital RNA modification that regulates mRNA stability, splicing, and nuclear export. As a key m^5^C reader, ALYREF promotes tumorigenesis across various cancers; however, its precise role in endometrial cancer (EC) remains unknown. ALYREF expression and clinical significance in EC were evaluated using bioinformatics and immunohistochemistry. In vitro and in vivo assays examined its functional roles. Integrated multi‐omics (RNA‐seq, RIP‐seq, and *m* ^5^C‐BS‐seq) and rescue assays identified downstream targets. RNA stability, nuclear‐cytoplasmic fractionation, site‐directed mutagenesis, dual‐luciferase, immunofluorescence, and Western blotting were performed to elucidate the underlying mechanisms. ALYREF is significantly upregulated in EC and serves as an independent prognostic factor for poor overall survival. Functional studies demonstrated that ALYREF markedly promotes EC cell proliferation, migration, invasion, and tumor growth. Mechanistically, *XRCC6* was identified as a direct functional target. ALYREF enhances *XRCC6* mRNA stability in an *m* ^5^C‐dependent manner, subsequently activating canonical Wnt/β‐catenin signaling to drive EC progression. Our findings reveal that ALYREF functions as a potential oncogenic driver in EC. The ALYREF–m^5^C–XRCC6 axis accelerates EC progression via Wnt/β‐catenin signaling, highlighting a promising therapeutic target.

## Graphical abstract

This study identifies ALYREF as an oncogenic m^5^C reader in endometrial cancer. ALYREF recognizes NSUN2‐mediated m^5^C modification on *XRCC6* mRNA to enhance transcript stability and increase XRCC6 expression. Elevated XRCC6 activates Wnt/β‐catenin signaling, resulting in accelerated tumor cell proliferation, migration, invasion, and xenograft tumor growth. Created with BioRender.com.

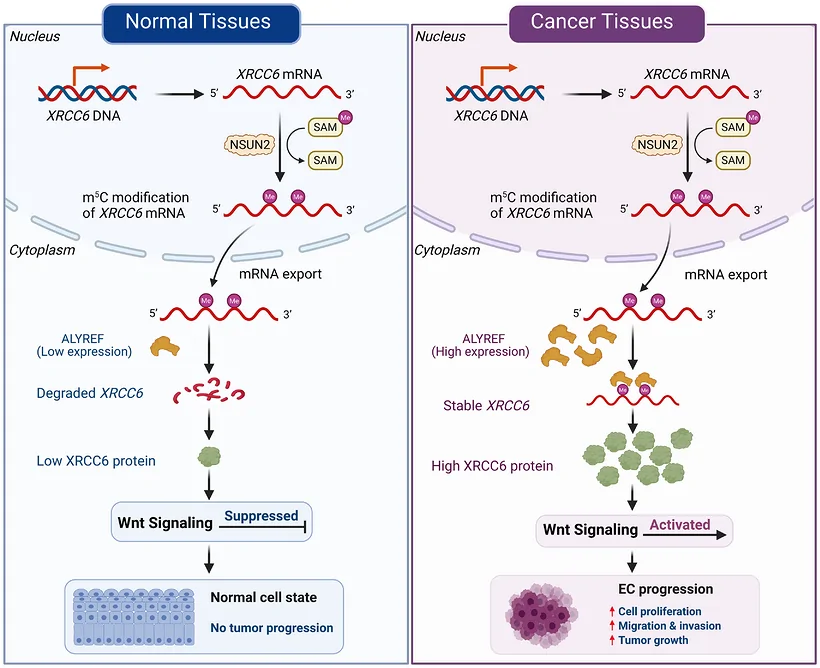

**Abbreviations**
ALYREF: Aly/REF export factor
CCK‐8: cell counting kit‐8
EC: endometrial cancer
EdU: 5‐Ethynyl‐2′‐deoxyuridine
GEO: Gene Expression Omnibus
HE: hematoxylin and eosin
IGV: Integrative Genomics Viewer
IHC: Immunohistochemistry
m^5^C: 5‐Methylcytosine
m^5^C‐BS‐seq: 5‐Methylcytosine Bisulfite Sequencing
NSUN2: NOP2/Sun RNA methyltransferase 2
NSUN6: NOP2/Sun RNA methyltransferase 6
RIP‐seq: RNA immunoprecipitation sequencing
RNA‐seq: RNA sequencing
RT‐qPCR: quantitative real‐time PCR
TCGA: The Cancer Genome Atlas
UCEC: Uterine Corpus Endometrial Carcinoma;
XRCC6: X‐ray repair cross‐complementing protein 6

## 1 Introduction

Endometrial cancer (EC) is one of the most common malignancies affecting the female reproductive system, and its global incidence has risen markedly in recent years [1]. Although approximately 90% of cases are detected at an early stage and achieve favorable 5‐year survival rates through standard treatments such as surgery and chemoradiotherapy [2], patients with locally advanced, recurrent, or metastatic disease often exhibit resistance to treatment and have a poor prognosis [3, 4]. Therefore, elucidating the molecular mechanisms underlying the development and progression of EC is crucial for identifying key driver genes and signaling pathways, thereby facilitating the discovery of novel diagnostic biomarkers and therapeutic targets.

Increasing evidence suggests that RNA post‐transcriptional modifications play crucial roles in the initiation and progression of cancer. To date, various dynamic RNA modifications have been identified, including N6‐methyladenosine, 5‐methylcytosine (m^5^C), and N1‐methyladenosine [5]. Among these, m^5^C is one of the most abundant and common post‐transcriptional modifications in eukaryotic mRNA, where it regulates RNA stability and nuclear export [6, 7]. Dysregulation of m^5^C modification and its regulatory factors has been closely associated with the development of various malignancies [8, 9]. As a dynamic and reversible process, m^5^C modification is regulated by a coordinated network of writers, erasers, and readers [10]. Aly/REF export factor (ALYREF), a well‐characterized m^5^C reader protein, recognizes and binds to m^5^C‐modified sites on mRNA, thereby regulating RNA metabolism [11, 12, 13]. Previous studies have demonstrated that ALYREF is highly expressed in certain malignancies and closely correlates with poor prognosis [14, 15]. By regulating the expression of oncogenes, ALYREF enhances tumor cell proliferation, migration, and invasion, while suppressing apoptosis [16, 17, 18, 19].

However, despite the growing recognition of the oncogenic roles of ALYREF in several malignancies, its biological function and molecular mechanism in EC remain largely unknown. In particular, it is still unclear whether an m^5^C‐mediated regulatory network exists in EC, what downstream targets are regulated by ALYREF, and through which signaling pathways ALYREF exerts its oncogenic effects. Addressing these questions may provide novel insights into the molecular mechanisms underlying EC progression and facilitate the identification of novel prognostic biomarkers and therapeutic targets. Therefore, the present study aims to investigate the biological function of ALYREF in EC, elucidate the underlying molecular mechanisms by which ALYREF promotes tumor progression, and evaluate its potential clinical significance.

## 2 Results

### 2.1 ALYREF Is Upregulated and Associated with Poor Prognosis in EC

**FIGURE 1:**
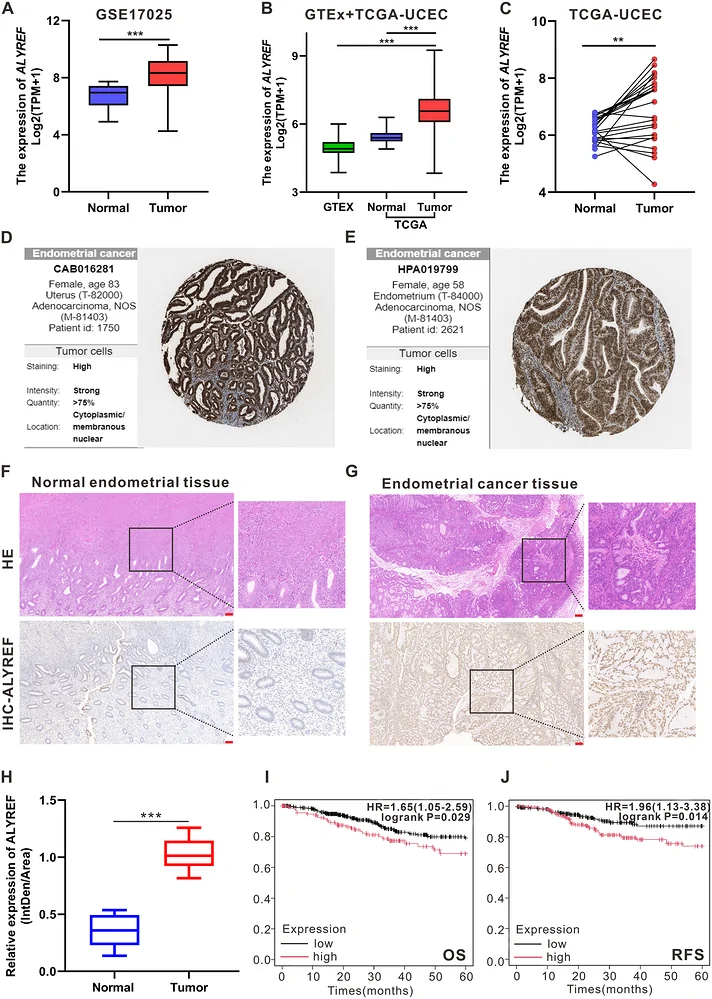
ALYREF is upregulated in EC and associated with poor prognosis. (A–C) Analysis of *ALYREF* expression in endometrial cancer (UCEC) datasets: (A) *ALYREF* mRNA expression in normal and tumor tissues from the GSE17025 dataset. (B) Expression of *ALYREF* across GTEx normal control tissues, TCGA normal endometrial tissues, and TCGA‐UCEC tumor tissues. (C) Paired‐sample comparison of *ALYREF* expression between adjacent normal and TCGA‐UCEC tumor tissues. (D,E) Representative immunohistochemistry (IHC) staining images of ALYREF protein in endometrial cancer from the Human Protein Atlas (HPA) database. (F,G) Representative H&E and IHC staining images of ALYREF in normal endometrial tissues (F) (*n* = 20) and endometrial cancer tissues (G) (*n* = 50). (H) Quantification of relative ALYREF protein expression (IntDen/Area) between normal and EC tissues based on IHC analysis. (I,J) Kaplan–Meier survival analysis of Overall Survival (OS) (I) and Relapse‐Free Survival (RFS) (J) according to *ALYREF* expression levels using the Kaplan–Meier Plotter database (TCGA‐UCEC cohort). *p < 0.05, **p < 0.01, ***p < 0.001; scale bars = 100 µm.

To investigate the role of ALYREF in EC, its expression levels were first analyzed using multiple independent public databases, including GEO, TCGA, GTEx, and HPA datasets. As shown in Figure 1A, analysis of the GEO dataset(GSE17025) revealed significantly elevated *ALYREF* expression in tumor tissues compared with normal endometrial tissues (*p* < 0.001). To further validate these findings, integrated analysis of TCGA‐UCEC and GTEx datasets demonstrated that *ALYREF* expression was markedly increased in tumor tissues relative to both GTEx normal uterine tissues and TCGA adjacent normal tissues (*p* < 0.001; Figure 1B). Moreover, paired‐sample analysis of TCGA‐UCEC cohort confirmed that *ALYREF* expression was significantly higher in tumor tissues than in matched adjacent normal tissues (*p* < 0.01; Figure 1C). Consistently, immunohistochemical data from The Human Protein Atlas database confirmed elevated ALYREF protein expression in EC tissues (Figure 1D,E).

To validate these observations in clinical specimens, paraffin‐embedded sections from 50 EC tissues and 20 normal endometrial tissues were subjected to hematoxylin and eosin (H&E) staining for histological evaluation and to IHC analysis for ALYREF expression. Representative H&E and IHC staining images revealed that ALYREF protein levels were markedly higher in EC tissues than in normal endometrial tissues (Figure 1F,G). Quantitative analysis of IHC staining further confirmed a significant increase in ALYREF protein levels in EC tissues (Figure 1H).

Furthermore, survival analysis using the Kaplan‐Meier Plotter database (TCGA‐UCEC cohort) revealed that patients with high ALYREF expression had significantly shorter overall survival (OS, *p* = 0.029) and relapse‐free survival (RFS, *p* = 0.014) than those with low ALYREF expression (Figure 1I,J). To further assess the prognostic value of ALYREF, Cox regression analyses were performed using the TCGA‐UCEC cohort. Univariate Cox regression analysis revealed that ALYREF expression was significantly associated with poorer OS. Moreover, multivariate Cox regression analysis confirmed that ALYREF remained an independent prognostic factor after adjustment for relevant clinicopathological variables (Table S1).

We next evaluated the association between ALYREF expression and clinicopathological characteristics in the TCGA‐UCEC cohort. ALYREF expression showed no significant association with clinical stage, although a gradual increase was observed in advanced‐stage tumors (Figure S1A). In contrast, ALYREF expression was significantly associated with histological grade, with higher expression observed in grade 3 tumors compared with grade 1 and 2 tumors (Figure S1B). Moreover, ALYREF expression differed among histological subtypes, with serous and mixed tumors exhibiting higher ALYREF levels than endometrioid tumors (Figure S1C). Collectively, these findings indicate that elevated ALYREF expression is associated with aggressive histological features and unfavorable clinical outcomes inEC.

### 2.2 ALYREF Promotes the Proliferation of EC Cells

**FIGURE 2:**
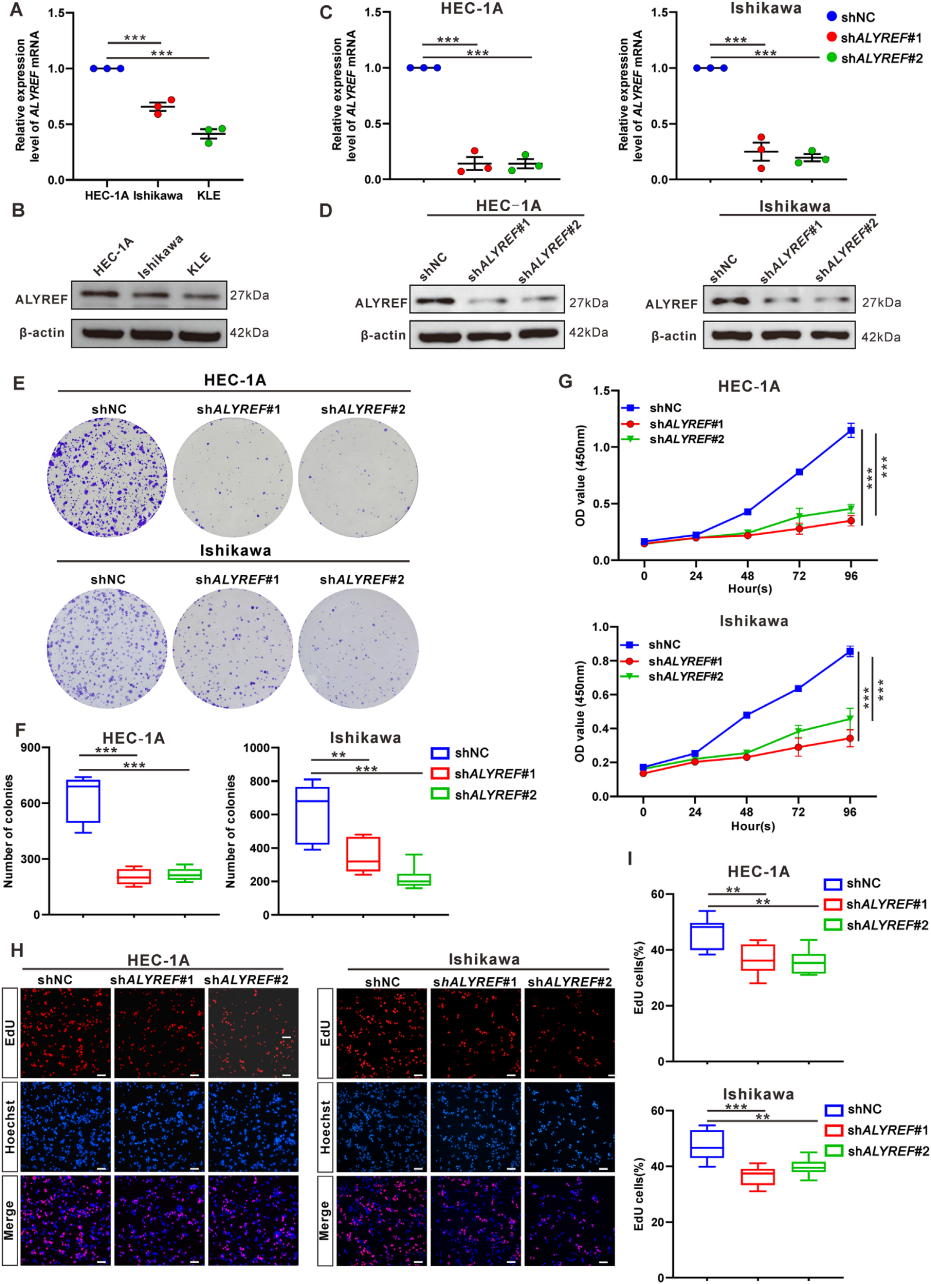
ALYREF knockdown inhibits the proliferation of EC cells. (A,B) RT‐qPCR (A) and Western blot (B) analyses showing *ALYREF* expression in HEC‐1A, Ishikawa, and KLE cells. (C,D) Knockdown efficiency of ALYREF in HEC‐1A and Ishikawa cells transduced with shNC, sh*ALYREF*#1, or sh*ALYREF*#2 was validated by RT‐qPCR (C) and western blotting (D). (E,F) Representative images (E) and quantitative analysis (F) of colony formation assays in control and *ALYREF*‐knockdown HEC‐1A and Ishikawa cells. (E,F) Representative images (E) and quantitative analysis (F) of colony formation assays in control and *ALYREF*‐knockdown HEC‐1A and Ishikawa cells. (G) Cell proliferation evaluated by CCK‐8 assays in control and *ALYREF*‐knockdown HEC‐1A and Ishikawa cells over 96 h. (H, I) Representative fluorescence images (H) and quantification (I) of EdU incorporation assays (EdU, red; Hoechst, blue) in control and *ALYREF*‐knockdown HEC‐1A and Ishikawa cells. *p < 0.05, **p < 0.01, ***p < 0.001; scale bars, 100 µm.

To investigate the biological function of ALYREF in EC cells, we first examined its endogenous expression levels in different EC cell lines. RT‐qPCR and western blotting analyses revealed that ALYREF exhibited relatively high expression levels in HEC‐1A and Ishikawa cells, whereas its expression was low in KLE cells (Figure 2A,B). Therefore, HEC‐1A and Ishikawa cell lines were selected for loss‐of‐function experiments. Stable ALYREF‐knockdown cell lines were established via lentivirus‐mediated shRNA transduction, and knockdown efficiency was successfully validated at both mRNA and protein levels (Figure 2C,D). Colony formation assays showed that ALYREF knockdown significantly reduced the number of colonies formed in both HEC‐1A and Ishikawa cells (Figure 2E,F). Consistently, CCK‐8 assays revealed that ALYREF knockdown markedly suppressed cell proliferation over time compared with control cells (Figure 2G). Furthermore, EdU incorporation assays demonstrated that the proportion of proliferating cells was significantly decreased following ALYREF knockdown (Figure 2H,I).

To further validate the pro‐proliferative role of *ALYREF*, gain‐of‐function experiments were performed in KLE cells, which exhibited low endogenous ALYREF expression. Successful ALYREF overexpression was confirmed by RT‐qPCR and western blotting analyses (Figure S2A,B). CCK‐8 assays showed that ALYREF‐overexpressing cells exhibited significantly enhanced proliferative capacity compared with vector control cells (Figure S2C). In addition, representative images from colony formation assays revealed increased colony‐forming capability following ALYREF overexpression, as confirmed by quantitative analysis (Figure S2D,E). Consistently, EdU staining showed that ALYREF overexpression increased the proportion of EdU‐positive proliferating cells (Figure S2F,G). Collectively, these loss‐ and gain‐of‐function studies demonstrate that ALYREF positively regulates EC cell proliferation.

### 2.3 ALYREF Promotes Migration, Invasion, and Tumor Growth of EC Cells

**FIGURE 3:**
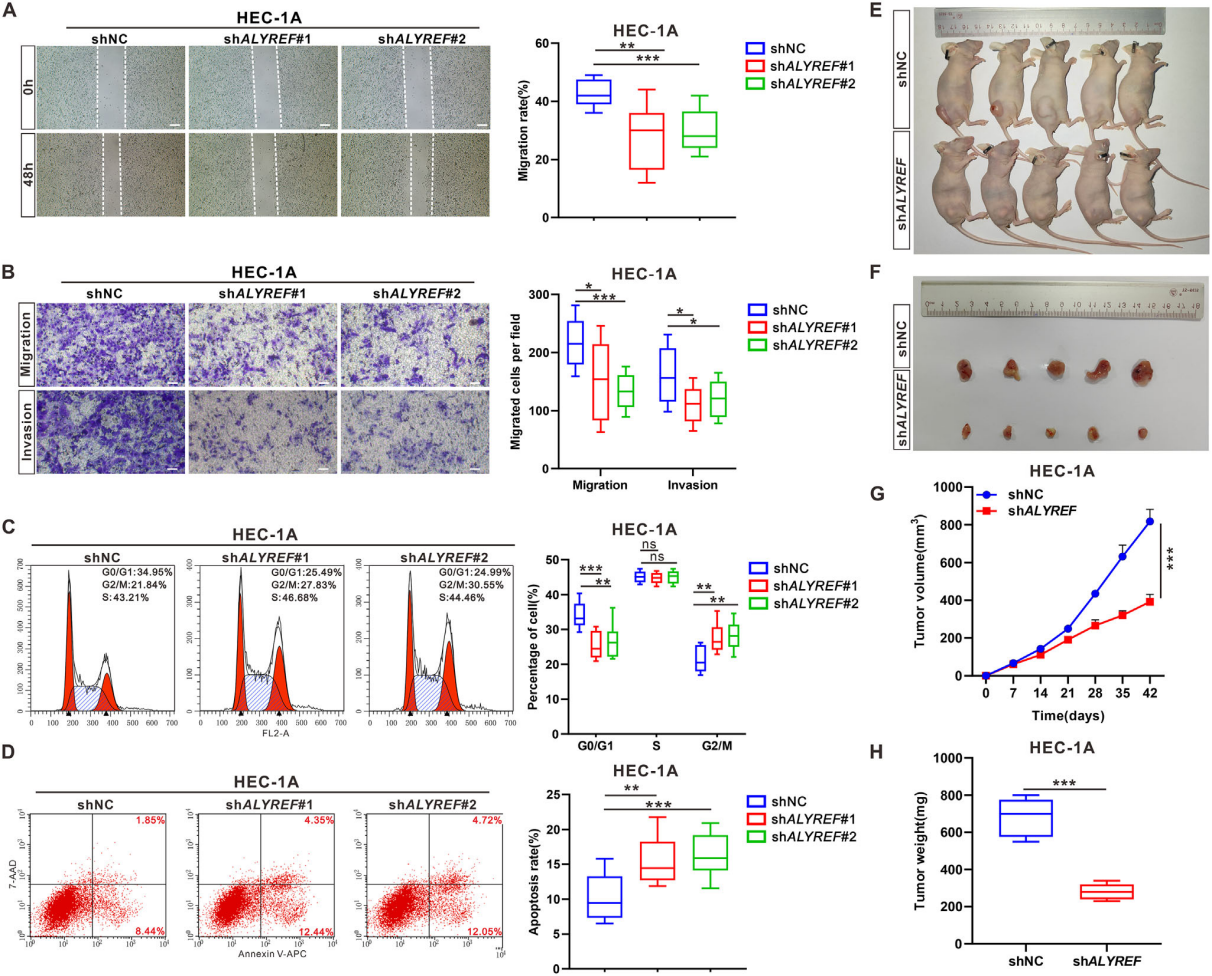
ALYREF knockdown inhibits the migration, invasion, and tumor growth of EC cells. (A) Representative images and quantification of wound healing assays in control and *ALYREF*‐knockdown HEC‐1A cells at 0 h and 48 h. (B) Representative images and quantification of Transwell migration and invasion assays in control and ALYREF‐knockdown HEC‐1A cells. (C) Cell cycle analysis by flow cytometry showing cell phase distribution (G0/G1, S, G2/M) in control and ALYREF‐knockdown HEC‐1A cells. (D) Apoptosis analysis by flow cytometry showing the percentage of apoptotic cells (Annexin V‐APC / 7‐AAD) in control and ALYREF‐knockdown HEC‐1A cells. (E) Representative images of tumor‐bearing nude mice at the end of the experiment. (F) Images of excised xenograft tumors from the shNC and shALYREF groups (*n* = 5 per group). (G) Tumor growth curves showing xenograft tumor volume (mm^3^) measured every 7 days over 42 days. (H) Quantitative comparison of final xenograft tumor weights (mg) between the shNC and sh*ALYREF* groups. *p < 0.05, **p < 0.01, ***p < 0.001, ns: not significant; scale bars = 100 µm.

To further characterize the oncogenic role of *ALYREF* in EC cells, we examined its effects on cell migration, invasion, cell cycle progression, and apoptosis. Wound healing assays demonstrated that ALYREF knockdown markedly delayed wound closure in HEC‐1A cells compared with shNC control cells (Figure 3A). Consistently, Transwell assays revealed that ALYREF knockdown impaired both migratory and invasive capacities of EC cells (Figure 3B). Flow cytometry analysis was subsequently performed to determine whether ALYREF regulates cell cycle progression and apoptosis. ALYREF knockdown resulted in a significant alteration of cell cycle distribution, characterized by a reduced G0/G1 population and G2/M phase arrest (Figure 3C). Moreover, apoptosis analysis showed that ALYREF knockdown significantly increased the proportion of apoptotic cells compared with control cells (Figure 3D). Similar results were observed in Ishikawa cells, where ALYREF knockdown significantly suppressed cell migration and invasion, induced G2/M phase arrest, and promoted apoptosis (Figure S3A–D), further supporting the oncogenic role of *ALYREF* in EC cells.

To further validate these findings, gain‐of‐function experiments were performed in KLE cells. ALYREF overexpression significantly enhanced migration and invasion capacities, as demonstrated by wound healing and Transwell assays (Figure S3E,F). In addition, ALYREF overexpression accelerated cell cycle progression and decreased apoptosis compared with vector control cells (Figure S3G,H).

Finally, the role of ALYREF in tumor growth was evaluated using a subcutaneous xenograft mouse model. HEC‐1A cells with stable ALYREF knockdown were subcutaneously injected into the flanks of nude mice. ALYREF knockdown significantly suppressed xenograft tumor growth in vivo, as evidenced by representative images of tumor‐bearing mice and excised xenograft tumors, as well as significantly reduced tumor volume and weight (Figure 3E–H).

### 2.4 Identification of the *ALYREF* Target Transcripts in EC

**FIGURE 4:**
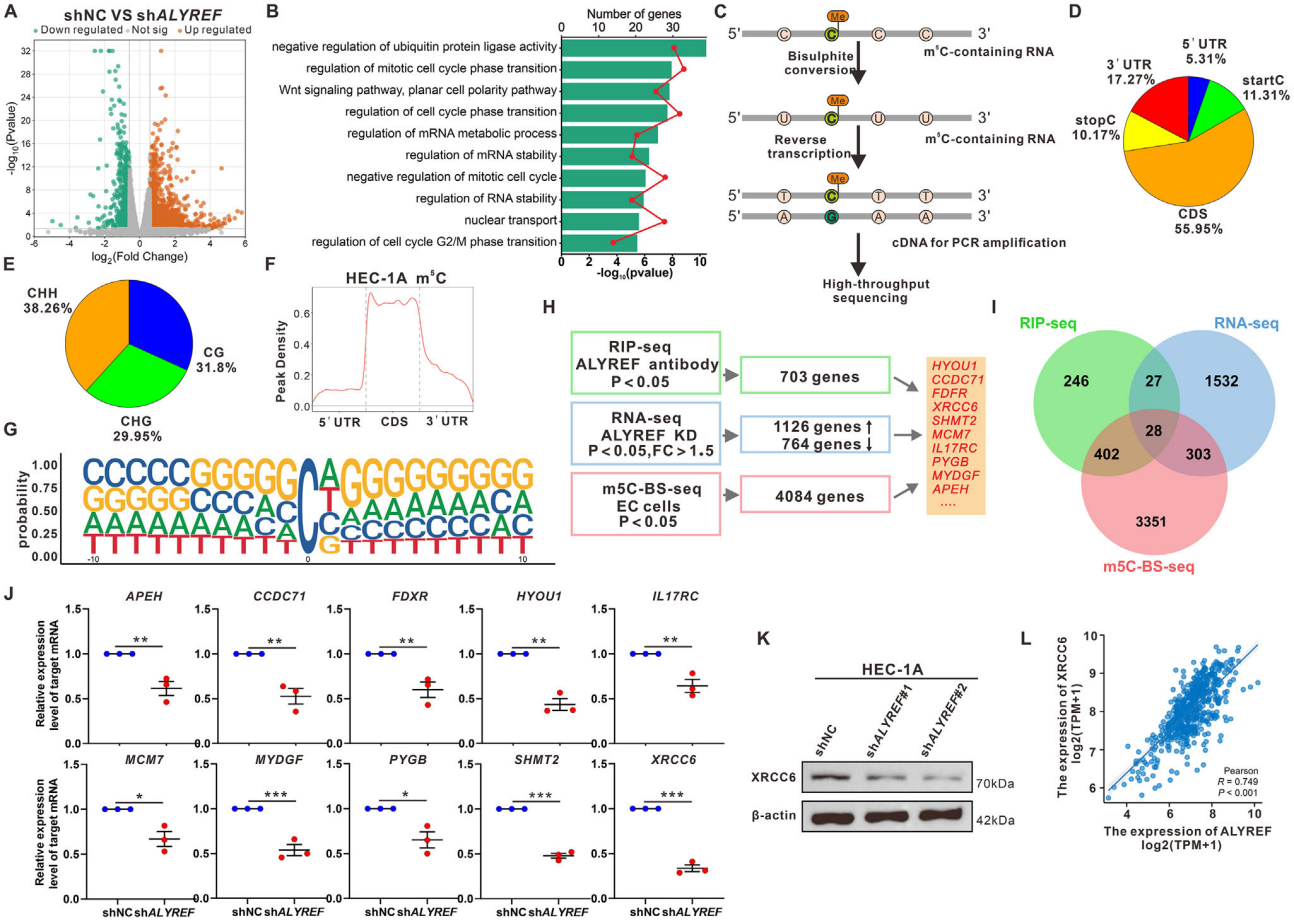
Identification of ALYREF candidate target transcripts in EC. (A) Volcano plot displaying DEGs identified by RNA‐seq in control and *ALYREF*‐knockdown HEC‐1A cells. (B) GO enrichment analysis of *ALYREF*‐bound transcripts identified via RIP‐seq in HEC‐1A cells. (C) Schematic diagram illustrating the experimental workflow of m^5^C‐BS‐seq. (D) Distribution of m^5^C sites across different RNA regions. (E) Proportions of CHG, CHH, and CG sequence contexts at m^5^C sites. (F) Density plot showing m^5^C peak distribution across 5′ UTR, CDS, and 3′ UTR regions in HEC‐1A cells. (G) Sequence logo representing the consensus motif enriched around m^5^C sites. (H) Schematic overview of the integrated downstream analysis of RNA‐seq, RIP‐seq, and m^5^C‐BS‐seq datasets. (I) Venn diagram illustrating overlapping transcripts identified across RNA‐seq, RIP‐seq, and m^5^C‐BS‐seq datasets. (J) Relative mRNA expression levels of the top 10 candidate target transcripts (*APEH, CCDC71, FDXR, HYOU1, IL17RC, MCM7, MYDGF, PYGB, SHMT2, XRCC6*) in HEC‐1A cells infected with shNC or sh*ALYREF* measured by RT‐qPCR. (K) Western blotting showing XRCC6 expression after *ALYREF* knockdown in HEC‐1A cells. (L) Pearson correlation analysis of *ALYREF* and *XRCC6* mRNA in EC tissues based on the TCGA‐UCEC database(*R = *0.749, *p*<0.001). *p < 0.05, **p < 0.01, ***p < 0.001.

To elucidate the molecular mechanisms underlying ALYREF‐mediated EC progression, RNA‐seq was first performed in HEC‐1A cells with ALYREF knockdown. A total of 1890 differentially expressed genes (DEGs) were identified (*p*<0.05, |FC|>1.5), including 1126 upregulated and 764 downregulated genes (Figure 4A). RIP‐seq analysis of HEC‐1A cells identified 703 transcripts enriched in ALYREF immunoprecipitates (*p*<0.05). GO enrichment analyses indicated that these ALYREF‐associated mRNAs were predominantly involved in cell cycle regulation, Wnt signaling, and RNA metabolism (Figure 4B). To characterize the global m^5^C modification landscape in EC cells, m^5^C‐BS‐seq was conducted in HEC‐1A cells, identifying 4,084 m^5^C‐modified transcripts (Figure 4C). These modifications were primarily enriched within the coding sequence (CDS), accounting for approximately 50%–60% of total sites, with lower frequencies in the 5′UTR and 3′UTR (Figure 4D). In addition, m^5^C sites were mainly distributed in CHH (38.26%) and CG (31.80%) contexts (Figure 4E), with high density across the CDS region (Figure 4F). Motif analysis further revealed conserved consensus sequence motifs enriched around the m^5^C‐modified sites (Figure 4G).

By integrating the RNA‐seq, RIP‐seq, and m^5^C‐BS‐seq datasets, 28 candidate transcripts were identified, comprising 22 downregulated and 6 upregulated transcripts (Figure 4H,I). Given that ALYREF is known to regulate mRNA nuclear export and stability, the top 10 candidates showing the most significant downregulation following ALYREF knockdown were selected for RT‐qPCR validation. In HEC‐1A cells, ALYREF knockdown led to varying degrees of downregulation in all 10 candidate transcripts, with XRCC6 showing the most significant decrease (Figure 4J). Consistently, western blotting further confirmed a marked reduction in XRCC6 protein expression following ALYREF knockdown in both HEC‐1A (Figure 4K) as well as Ishikawa cells (Figure S4A). Immunofluorescence analysis further demonstrated that ALYREF knockdown significantly decreased XRCC6 fluorescence intensity in both cell lines (Figure S4B,C). In addition, correlation analysis based on the TCGA‐UCEC cohort revealed a strong positive correlation between *ALYREF* and *XRCC6* mRNA expression levels (*R* = 0.749, *p < *0.001, Figure 4L). At the protein level, immunohistochemical staining was performed to assess ALYREF and XRCC6 protein expression in 50 EC tissues and 20 normal endometrial tissues (Figure S4D,E). H‐score‐based quantitative analysis of IHC staining revealed a significant positive correlation between ALYREF and XRCC6 protein levels in clinical samples, as assessed by Spearman correlation analysis (*R* = 0.82, *p* < 0.001, Figure S4F).

### 2.5 XRCC6 Overexpression Rescues the Inhibitory Effects of ALYREF Knockdown on EC Progression

**FIGURE 5:**
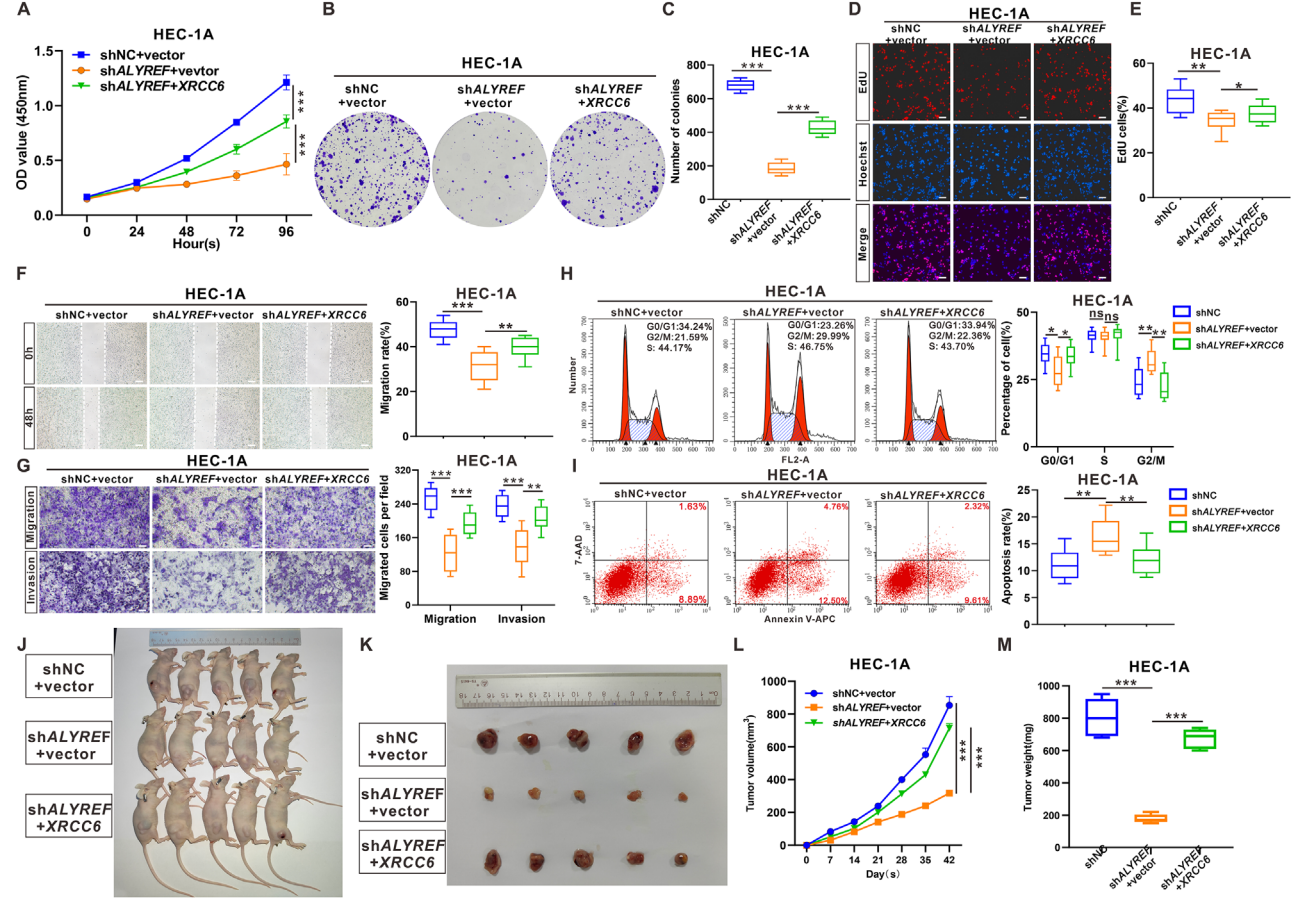
XRCC6 overexpression rescues the inhibitory effects of ALYREF knockdown on HEC‐1A cell proliferation, metastasis, and tumor growth in vivo. (A) CCK‐8 assay demonstrating that *XRCC6* overexpression restores cell viability in *ALYREF*‐knockdown HEC‐1A cells.(B,C) Colony formation assay assessing the rescue effect of XRCC6 overexpression on HEC‐1A cell proliferation, with (B) representative colony images and (C) statistical quantification of colony numbers.(D,E) EdU incorporation assay evaluating DNA synthesis, showing (D) representative immunofluorescence images (EdU in red and Hoechst in blue) and (E) quantification of the percentage of EdU‐positive cells.(F) Wound healing assay and quantitative analysis of migration rates assessing the impact of *XRCC6* overexpression on cell migration in *ALYREF*‐knockdown HEC‐1A cells.(G) Transwell assays and corresponding quantification of migrated and invaded cells in HEC‐1A cells under different treatment conditions. (H) Cell cycle analysis by PI staining and flow cytometry, with quantification of cells across G0/G1, S, and G2/M phases. (I) Apoptosis analysis using Annexin V/7‐AAD staining and flow cytometry, with statistical quantification of apoptotic rates. (J–M) Subcutaneous xenograft tumor model in nude mice (*n* = 5 per group) showing the in vivo rescue effects. (J) Photograph of mice from the shNC+vector, sh*ALYREF*+vector, and sh*ALYREF*+*XRCC6* groups. (K) Photograph of the harvested xenograft tumors. (L) Tumor growth curves measuring tumor volumes over 42 days. (H) Statistical analysis of the final tumor weights (mg). *p < 0.05, **p < 0.01, ***p < 0.001; ns: not significant; scale bars = 100 µm.

To determine whether XRCC6 mediates the oncogenic effects of ALYREF in EC cells, rescue experiments were performed. Colony formation, CCK‐8, and EdU incorporation assays demonstrated that XRCC6 overexpression rescued the impaired proliferative capacity caused by ALYREF knockdown (Figures 5A–E and S5A–E). Similarly, wound healing and Transwell assays revealed that XRCC6 overexpression restored the migratory and invasive capacities suppressed by ALYREF knockdown (Figures 5F,G and S5F,G). Cell cycle analysis showed that XRCC6 overexpression alleviated the G2/M arrest induced by ALYREF knockdown (Figures 5H and S5H). Furthermore, apoptosis assays indicated that XRCC6 overexpression reversed the increased apoptosis induced by ALYREF knockdown (Figures 5I and S5I).

To further validate the role of the ALYREF‐XRCC6 axis in vivo, xenograft experiments were conducted in nude mice. Consistent with the in vitro findings, XRCC6 overexpression markedly reversed the tumor‐suppressive effect of ALYREF knockdown, as evidenced by increased tumor volumes and weights (Figure 5J–M).

### 2.6 ALYREF Enhances *XRCC6* mRNA Stability

Previous integrated analysis of m^5^C‐BS‐seq and RIP‐seq datasets revealed that *XRCC6* mRNA contains both m^5^C modification sites and ALYREF‐binding signals, suggesting that *XRCC6* may represent a potential downstream target of ALYREF‐mediated RNA regulation. To validate the interaction between *ALYREF* and *XRCC6* mRNA, RNA immunoprecipitation followed by quantitative PCR (RIP‐qPCR) was performed in HEC‐1A cells using an anti‐ALYREF antibody. Compared with the IgG control, *XRCC6* mRNA was significantly enriched in ALYREF immunoprecipitates (Figure S6A), confirming the specific association between ALYREF and *XRCC6* transcripts.

**FIGURE 6:**
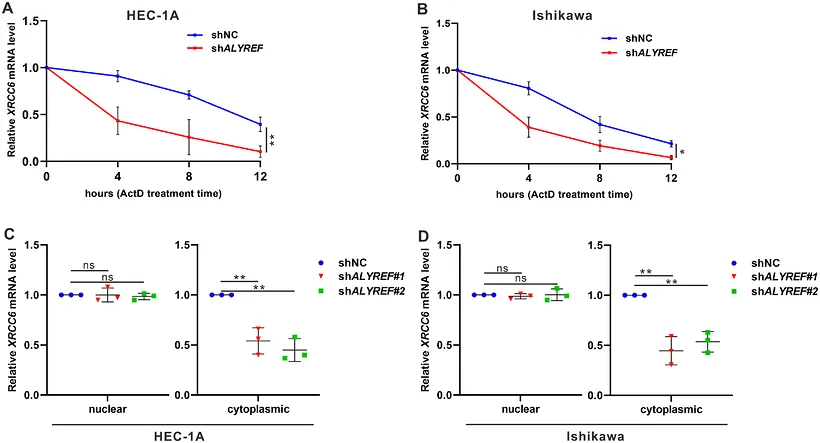
ALYREF stabilizes *XRCC6* mRNA in the cytoplasm of EC cells. (A,B) mRNA stability assay using Actinomycin D (ActD) treatment. The relative degradation rate and half‐life of *XRCC6* mRNA were determined by RT‐qPCR at the indicated time points (0, 4, 8, and 12 h) following ActD treatment inHEC‐1A (A) and Ishikawa (B)cells transfected with shNC or sh*ALYREF*. (C,D) Nuclear‐cytoplasmic fractionation analysis followed by RT‐qPCR quantification of *XRCC6* mRNA expression in the nuclear and cytoplasmic fractions of (C) HEC‐1A and (D) Ishikawa cells after *ALYREF* knockdown.*p < 0.05, **p < 0.01; ns: not significant.

As an RNA‐binding protein, ALYREF has been implicated in multiple aspects of RNA metabolism, including mRNA stability regulation and nuclear export [11, 20]. To determine whether ALYREF regulates *XRCC6* expression through modulation of mRNA stability, we performed Actinomycin D chase assays in HEC‐1A and Ishikawa cells. Following ALYREF knockdown, *XRCC6* mRNA exhibited accelerated degradation and a significantly reduced half‐life compared with control cells (Figure 6A,B), indicating that ALYREF contributes to *XRCC6* mRNA stabilization.

Because ALYREF also functions as a known regulator of mRNA nuclear export, we next examined whether altered XRCC6 expression resulted from impaired RNA subcellular localization. Nuclear and cytoplasmic RNA fractionation analysis showed that ALYREF knockdown markedly reduced cytoplasmic *XRCC6* mRNA levels, whereas nuclear *XRCC6* mRNA abundance remained largely unchanged (Figure 6C,D). These results indicate that ALYREF primarily regulates XRCC6 expression by maintaining cytoplasmic mRNA stability rather than by facilitating its nuclear export.

### 2.7 NSUN2‐Mediated m^5^C Modification Enables ALYREF‐Dependent XRCC6 Regulation

**FIGURE 7:**
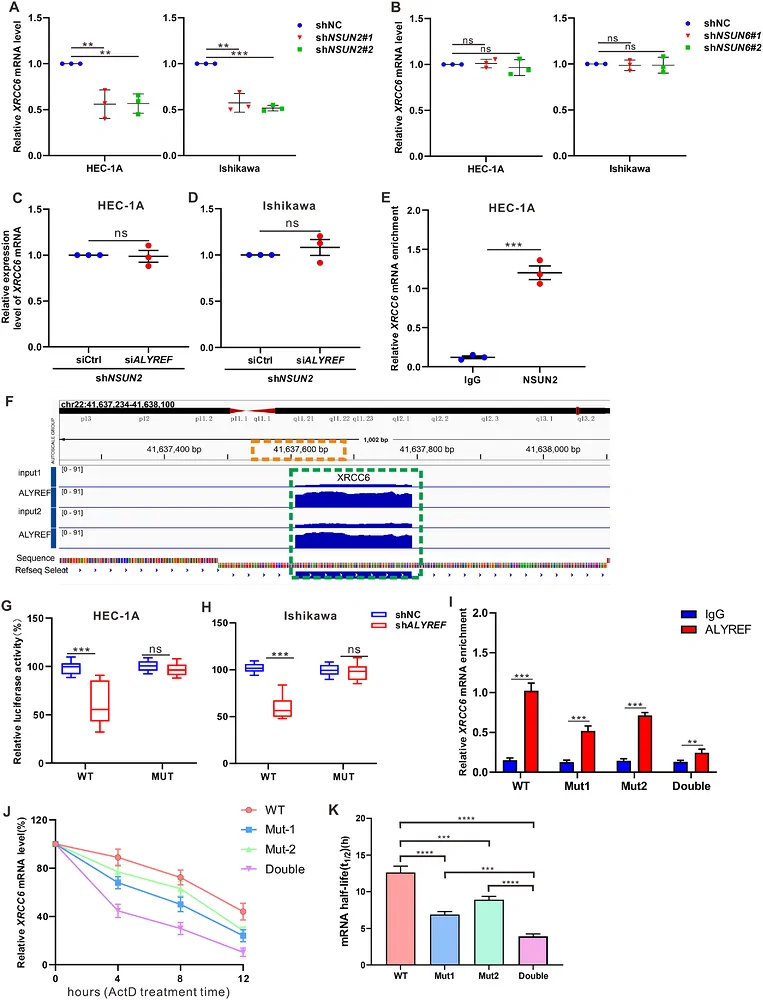
*ALYREF* regulates XRCC6 expression and transcript stability in an NSUN2‐mediated m^5^C‐dependent manner. (A,B) RT‐qPCR analysis of *XRCC6* mRNA expression levels in HEC‐1A and Ishikawa cells following the individual knockdown of (A) NSUN2 or (B) NSUN6. (C,D) RT‐qPCR analysis of *XRCC6* mRNA expression in HEC‐1A (C) and Ishikawa (D) cells with *NSUN2* knockdown co‐transfected with either control siRNA or ALYREF‐targeted siRNA. (E) RIP‐qPCR using anti‐NSUN2 antibody showed significant enrichment of *XRCC6* mRNA compared with IgG control in HEC‐1A cells. (F) IGV (Integrative Genomics Viewer) tracks of ALYREF RIP‐seq and corresponding input controls within the *XRCC6* mRNA locus, showing prominent ALYREF‐binding peaks (indicated by the dashed box). (G,H) Relative luciferase activity of XRCC6‐WT and XRCC6‐MUT reporters constructs measured in HEC‐1A (G) and Ishikawa (H) cells transfected with shNC or sh*ALYREF*. (I) ALYREF RIP‐qPCR analysis evaluating ALYREF enrichment on wild‐type (WT), single‐site mutated (Mut1 and Mut2), and double‐site mutated (Double) *XRCC6* transcripts. IgG served as the negative control. (J,K) Actinomycin D (ActD) chase assay evaluation of *XRCC6* WT and mutant constructs showing (J) the decay kinetics and (K) the statistical quantification of *XRCC6* mRNA half‐life (t_1/2_). *p < 0.05, **p < 0.01, ***p < 0.001; ns, not significant.

Given that ALYREF functions as an m^5^C reader and recognizes target RNAs in an m^5^C‐dependent manner, we next investigated the methyltransferase responsible for *XRCC6* mRNA modification and whether ALYREF‐mediated regulation depends on this modification. Previous studies have identified NOP2/Sun RNA methyltransferase 2 (NSUN2) and NOP2/Sun RNA methyltransferase 6 (NSUN6) as major mRNA m^5^C methyltransferases. Therefore, we examined whether NSUN2 or NSUN6 contributes to *XRCC6* regulation. *NSUN2* or *NSUN6* was individually knocked down in HEC‐1A and Ishikawa cells, and knockdown efficiency was confirmed by RT‐qPCR (Figure S7A,B). *XRCC6* mRNA expression was significantly reduced following NSUN2 knockdown, whereas NSUN6 knockdown had no obvious effect on XRCC6 expression (Figure 7A,B), suggesting that NSUN2, rather than NSUN6, is primarily involved in *XRCC6* mRNA regulation.

To determine whether ALYREF‐dependent regulation of *XRCC6* requires NSUN2‐mediated m^5^C modification, *ALYREF* was further knocked down in NSUN2‐knockdown cells. Under normal conditions, *ALYREF* knockdown significantly decreased *XRCC6* mRNA abundance. However, in the absence of *NSUN2*, additional knockdown of *ALYREF* failed to cause a further reduction in *XRCC6* mRNA levels (Figure 7C,D). These findings indicate that ALYREF‐mediated regulation of *XRCC6* depends on NSUN2‐associated m^5^C modification, suggesting that NSUN2‐derived m^5^C marks serve as a prerequisite for ALYREF recognition and stabilization of *XRCC6* transcripts. To further establish the association between NSUN2 and *XRCC6* mRNA, NSUN2 RIP‐qPCR was performed in HEC‐1A cells. Compared with IgG controls, *XRCC6* mRNA was significantly enriched in NSUN2 immunoprecipitates (Figure 7E), demonstrating an association between NSUN2 and *XRCC6* transcripts.

We next analyzed *XRCC6* m^5^C modification patterns using our m^5^C‐BS‐seq dataset. Visualization through Integrative Genomics Viewer (IGV) identified distinct m^5^C peaks within the *XRCC6* transcript region, which were consistent with m^5^C sites predicted by the RMVar 2.0 database (Figure S7C,D). Integration with RIP‐seq data further revealed that ALYREF‐binding regions overlapped with the identified m^5^C modification sites on *XRCC6* mRNA (Figure 7F), suggesting that ALYREF may recognize these m^5^C‐modified regions to regulate *XRCC6* transcript stability.

To determine whether these m^5^C sites are functionally required for ALYREF‐mediated *XRCC6* regulation, luciferase reporter constructs containing either wild‐type *XRCC6* sequences (XRCC6‐WT) or *XRCC6* sequences carrying mutations at the key m^5^C modification sites (XRCC6‐MUT) were generated (Figure S7E). ALYREF knockdown significantly reduced luciferase activity driven by XRCC6‐WT, whereas the activity of the XRCC6‐MUT reporter remained unchanged (Figure 7G,H), indicating that *XRCC6* m^5^C sites are essential for ALYREF‐dependent post‐transcriptional regulation.

To further define the contribution of individual m^5^C sites, two identified m^5^C sites were individually or simultaneously mutated to generate Mut1, Mut2, and Double Mut *XRCC6* constructs (Figure S7F). ALYREF RIP‐qPCR analysis demonstrated that mutation of either individual site partially reduced ALYREF enrichment, whereas simultaneous mutation of both sites caused a further marked reduction in ALYREF binding (Figure 7I). Consistently, Actinomycin D chase assays showed that single‐site mutations decreased *XRCC6* mRNA stability, while double‐site mutation resulted in a further reduction in transcript half‐life (Figure 7J,K). Together, these results demonstrate that the two m^5^C modification sites within *XRCC6* mRNA cooperatively facilitate ALYREF recognition and maintain *XRCC6* transcript stability.

### 2.8 ALYREF Activates Wnt/β‐Catenin Signaling Through Regulating XRCC6 Expression

**FIGURE 8:**
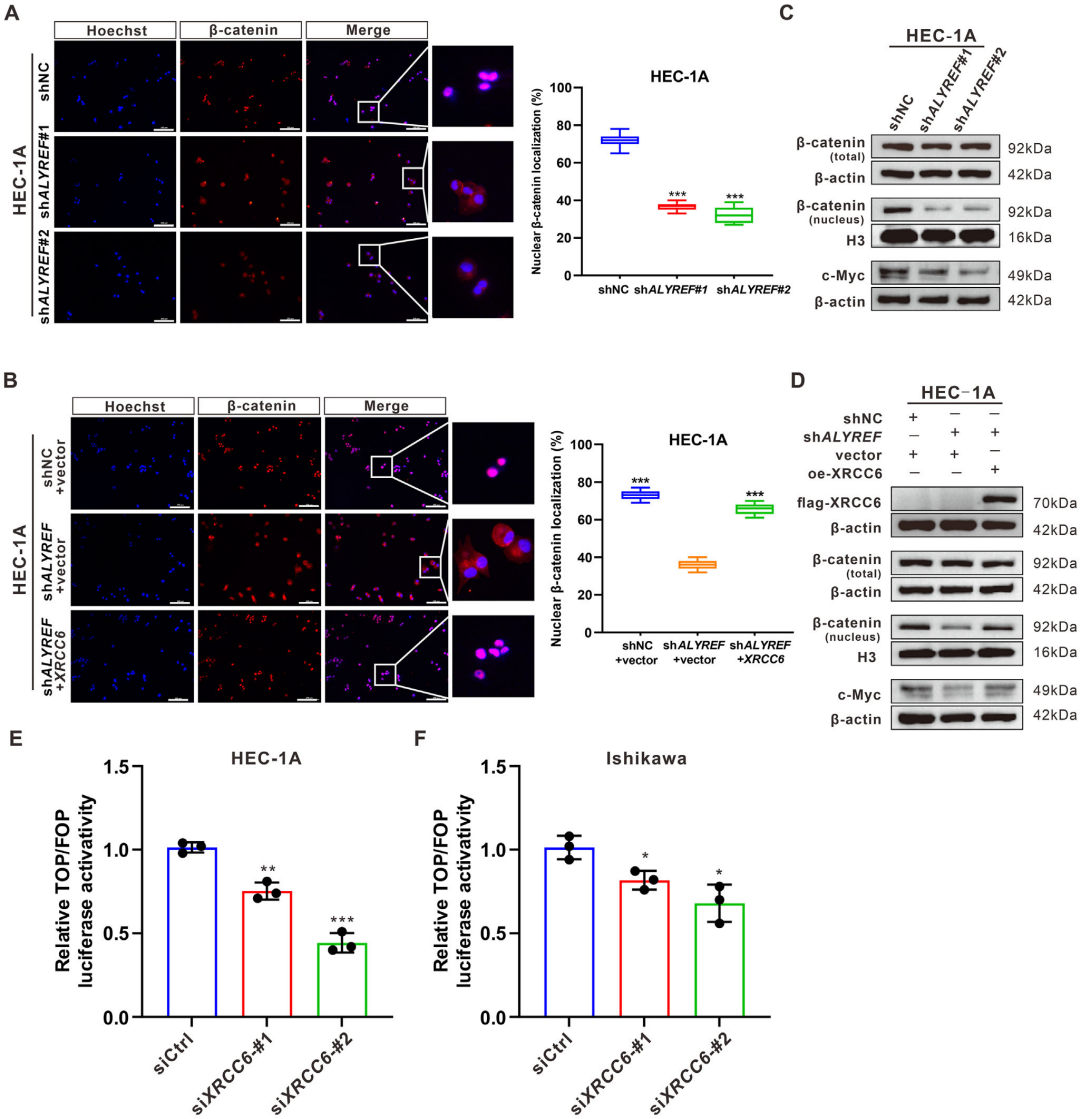
ALYREF activates canonical Wnt/β‐catenin signaling through regulating XRCC6 expression. (A) Representative immunofluorescence images and quantitative analysis showing β‐catenin subcellular localization in HEC‐1A cells after ALYREF knockdown. (B) Representative immunofluorescence images and quantitative analysis demonstrating that XRCC6 overexpression rescues β‐catenin nuclear translocation in ALYREF‐knockdown HEC‐1A cells. Nuclei were stained with Hoechst. Scale bars = 100 µm. (C) Western blot analysis of total β‐catenin, nuclear β‐catenin, and c‐Myc protein levels in HEC‐1A cells following ALYREF knockdown. β‐actin and Histone H3 (H3) were utilized as loading controls for the total and nuclear fractions, respectively. (D) Western blot analysis of flag‐XRCC6, total β‐catenin, nuclear β‐catenin, and c‐Myc expression in HEC‐1A cells transfected with shNC + vector, sh*ALYREF* + vector, or sh*ALYREF* + oe‐*XRCC6*. (E, F) Relative TOP/FOP flash luciferase reporter activity in HEC‐1A (E) and Ishikawa (F) cells transfected with control siRNA or siRNAs targeting XRCC6. *p < 0.05, ** *p* < 0.01, ***p < 0.001.

Previous studies have indicated that XRCC6 is associated with regulation of Wnt/β‐catenin signaling and contributes to multiple tumor‐related biological processes [21, 22]. In addition, GO enrichment analysis of ALYREF RIP‐seq targets revealed significant enrichment of the Wnt/β‐catenin signaling pathway (Figure 4B), suggesting that ALYREF may regulate Wnt signaling through XRCC6. Immunofluorescence analysis showed that ALYREF knockdown markedly reduced nuclear localization of β‐catenin in HEC‐1A cells, whereas XRCC6 overexpression restored β‐catenin nuclear localization (Figure 8A,B). Nuclear and cytoplasmic fractionation followed by western blotting further demonstrated that ALYREF knockdown significantly decreased nuclear β‐catenin and c‐Myc protein levels, while total β‐catenin levels remained largely unchanged (Figure 8C). Importantly, XRCC6 overexpression rescued the reduction of nuclear β‐catenin and c‐Myc expression induced by ALYREF knockdown (Figure 8D). Similar results were observed in Ishikawa cells, as confirmed by immunofluorescence staining and nuclear/cytoplasmic fractionation assays (Figure S8A–F).

To investigate whether XRCC6 regulates Wnt/β‐catenin signaling through direct interaction with β‐catenin, co‐immunoprecipitation (Co‐IP) assays were performed in HEC‐1A cells. Although XRCC6 was efficiently immunoprecipitated, no obvious enrichment of β‐catenin was detected in XRCC6 immunoprecipitates (Figure S8G), suggesting that XRCC6‐mediated regulation of Wnt/β‐catenin signaling may not require direct binding between XRCC6 and β‐catenin. We next evaluated β‐catenin/TCF transcriptional activity using TOP/FOP Flash reporter assays. XRCC6 knockdown significantly reduced TOP/FOP luciferase activity ratios in both HEC‐1A and Ishikawa cells (Figure 8E,F), demonstrating that XRCC6 promotes β‐catenin/TCF‐dependent transcriptional activity. Furthermore, ALYREF knockdown significantly reduced the expression of canonical Wnt target genes AXIN2 and CCND1 (Figure S8H–K), further supporting the role of the ALYREF/XRCC6 axis in regulating Wnt/β‐catenin signaling output.

To further determine whether the functional rescue mediated by XRCC6 depends on canonical Wnt/β‐catenin signaling, pathway‐inhibition rescue experiments were performed using the Wnt inhibitor XAV‐939. Functional assays, including CCK‐8 and colony formation assays, demonstrated that XRCC6 overexpression significantly restored the impaired proliferative ability caused by ALYREF knockdown, whereas XAV‐939 treatment markedly attenuated this rescue effect (Figure S9). Collectively, these findings demonstrate that ALYREF enhances canonical Wnt/β‐catenin signaling activity by regulating XRCC6 expression and promotes downstream transcriptional activation.

## 3 Discussion

As a key post‐transcriptional RNA modification, m^5^C is widely distributed across various RNA molecules and plays essential roles in regulating RNA subcellular localization, structural stability, alternative splicing, and metabolic fate [11, 23, 24]. In recent years, the epitranscriptomic dysregulation of m^5^C in tumorigenesis has attracted increasing attention. However, the precise molecular mechanisms by which specific m^5^C reader proteins mediate m^5^C‐dependent regulation of mRNA fate and oncogenic signaling in EC remain largely unexplored. ALYREF, one of the earliest identified mRNA m^5^C reader proteins, has been reported to promote tumor progression in various human malignancies. For instance, in nasopharyngeal carcinoma, ALYREF facilitates metastasis by binding to m^5^C‐modified *NOTCH1* mRNA and enhancing its expression [25]. In low‐grade glioma, ALYREF acts as an m^5^C reader to synergize with the m^5^C writer NSUN2, thereby significantly promoting glioma cell migration [19]. In non‐small cell lung cancer (NSCLC), ALYREF recognizes and stabilizes m^5^C‐modified *YAP1* mRNA, which in turn simultaneously activates both the Hippo and β‐catenin signaling pathways to drive NSCLC progression [26].

Although ALYREF has been established as an oncogenic m^5^C reader in multiple cancers, accumulating evidence indicates that its downstream regulatory network is highly tumor‐context dependent. Distinct ALYREF‐regulated transcriptomes have been identified across different cancer types, suggesting that ALYREF exerts its oncogenic functions through cancer‐specific downstream effectors rather than a single universal target gene network. In the present study, integrated analysis combining GTEx normal uterine tissues with TCGA‐UCEC datasets confirmed that ALYREF is significantly upregulated in EC tissues. Furthermore, multivariate Cox regression analysis established that high ALYREF expression serves as an independent prognostic factor for unfavorable overall survival in patients. Functional knockdown experiments in HEC‐1A and Ishikawa cells, complemented by gain‐of‐function rescue assays in low‐expressing KLE cells, collectively demonstrated that ALYREF markedly promotes EC cell proliferation, colony formation, migration, and invasion both in vitro and in vivo.

To delineate the EC‐specific downstream regulatory network of ALYREF, an unbiased integrative multi‐omics strategy was employed, combining RNA‐seq, RIP‐seq, and m^5^C‐BS‐seq datasets. Among the high‐confidence candidate targets screened, X‐ray repair cross‐complementing 6 (XRCC6) emerged as the most critical functional effector. The XRCC gene family plays a crucial role in the DNA damage response and the maintenance of genomic stability [27, 28]. Specifically, XRCC6, which encodes the Ku70 subunit of the non‐homologous end joining (NHEJ) repair complex, is frequently upregulated in various human cancers [29, 30, 31, 32, 33, 34]. Importantly, XRCC6 was not selected based on prior assumptions or candidate‐gene approaches; rather, it emerged naturally from this data‐driven multi‐omics workflow. Subsequent functional rescue experiments confirmed that restoration of XRCC6 expression effectively reversed the inhibitory effects of ALYREF knockdown on EC cell proliferation, migration, and invasion. Furthermore, immunohistochemical (IHC) co‐analysis based on the H‐score evaluation system in clinical patient specimens revealed a significant positive protein‐level correlation between ALYREF and XRCC6, providing direct clinical evidence for the functional activity of this regulatory axis in human EC tissues.

Mechanistically, our study elucidates the specific manner in which ALYREF regulates the metabolic fate of *XRCC6* mRNA. ALYREF knockdown significantly accelerated the degradation rate of *XRCC6* mRNA without altering its nucleocytoplasmic distribution ratio, confirming that ALYREF regulates XRCC6 expression primarily by enhancing mRNA stability rather than affecting its nuclear export. At the epitranscriptomic level, RIP‐qPCR assays demonstrated that *XRCC6* mRNA was closely associated with NSUN2, the primary m^5^C methyltransferase. Knockdown of NSUN2 not only reduced *XRCC6* mRNA stability but also abolished the regulatory effect of ALYREF knockdown on *XRCC6* mRNA, demonstrating that ALYREF‐mediated regulation depends on NSUN2‐catalyzed m^5^C modification. Crucially, to clarify the core binding site mechanism, we mapped two adjacent m^5^C sites within the coding region of *XRCC6* mRNA and constructed single‐ and double‐site mutant vectors. RIP‐qPCR and actinomycin D half‐life assays revealed that mutating a single m^5^C site only partially impaired ALYREF binding capacity and mRNA stability, whereas mutating both sites simultaneously resulted in a synergistic and significant loss of ALYREF binding affinity and mRNA half‐life. These findings indicate that these two adjacent m^5^C sites may function cooperatively as a local m^5^C recognition module, although further structural biology and biochemical studies are required to fully validate this model.

Investigation into downstream signaling pathways revealed that the ALYREF/XRCC6 axis critically engages the canonical Wnt/β‐catenin pathway. TOP/FOP Flash dual‐luciferase reporter assays confirmed that *XRCC6* knockdown significantly suppressed β‐catenin/TCF transcriptional activity. Although Co‐IP assays revealed no direct physical interaction between XRCC6 and β‐catenin, XRCC6 markedly promoted the nuclear accumulation of β‐catenin and activated the expression of its canonical downstream target genes, including *AXIN2*, *CCND1* (*Cyclin D1*), and *MYC*. Because XRCC6 is a multifunctional nuclear protein, it likely regulates β‐catenin transcriptional output indirectly (e.g., by modulating nuclear co‐activator complex assembly or altering local chromatin accessibility). Importantly, treatment with the canonical Wnt inhibitor XAV‐939 significantly blocked the proliferative and invasive rescue conferred by XRCC6 overexpression in ALYREF‐deficient cells. This chemical biology evidence confirms that the oncogenic phenotype driven by the ALYREF/XRCC6 axis is functionally dependent on canonical Wnt/β‐catenin signaling activation.

From a clinical translational perspective, our findings indicate that the ALYREF–m^5^C–XRCC6 axis serves as a potential prognostic biomarker and therapeutic target in EC. Given the dual roles of XRCC6 in both NHEJ DNA damage repair and Wnt signaling, patients with elevated ALYREF/XRCC6 expression may potentially benefit from targeted therapies directed against the Wnt/β‐catenin pathway or DNA damage repair pathways.

Several limitations of this study should be acknowledged: First, our clinical protein‐level validation was based on a single‐center retrospective cohort with a relatively modest sample size; larger prospective multicenter cohorts are needed to further establish its independent prognostic value. Second, although the cell line models used (Ishikawa, HEC‐1A, KLE) represent differential endogenous ALYREF expression levels, they do not fully encapsulate the genomic heterogeneity across TCGA‐defined molecular subtypes (such as *POLE*‐ultramutated, MMRd, or *TP53*‐abnormal). Third, while mechanistic validation was established using shRNA and overexpression plasmid systems, future studies utilizing conditionally inducible CRISPR‐Cas9 knockout models, primary EC cells, and patient‐derived organoid (PDO) models will provide more compelling translational validation. Fourth, this study primarily focused on cell‐autonomous regulation; whether the ALYREF/XRCC6 axis participates in non‐cell‐autonomous interactions with tumor microenvironment (TME) components–‐such as cancer‐associated fibroblasts (CAFs) and tumor‐infiltrating immune cells–‐remains to be thoroughly explored in future work.

## 4 Conclusions

In summary, ALYREF is significantly upregulated in endometrial cancer tissues, and its elevated expression independently correlates with poor patient prognosis. ALYREF promotes EC cell proliferation, migration, invasion, cell cycle progression, and tumor growth both in vitro and in vivo. Mechanistically, ALYREF recognizes m^5^C sites deposited by *NSUN2* on *XRCC6* mRNA, thereby enhancing *XRCC6* mRNA stability and subsequently activating the canonical Wnt/β‐catenin signaling pathway to drive tumor progression. These findings identify a novel ALYREF–m^5^C–XRCC6–Wnt/β‐catenin oncogenic axis in endometrial cancer, broaden our understanding of the tumor‐context‐dependent epitranscriptomic regulatory network of ALYREF, and provide a theoretical foundation for the development of precision diagnostic and epitranscriptomic therapeutic strategies targeting this signaling axis.

## 5 Materials and Methods

### 5.1 Public Database Analysis

Publicly available databases were used to evaluate the expression pattern, protein expression, clinicopathological significance, and prognostic value of ALYREF in endometrial cancer. The gene expression profile of the GSE17025 dataset was obtained from the Gene Expression Omnibus (GEO) database (https://www.ncbi.nlm.nih.gov/geo/) and used to compare ALYREF mRNA expression between normal endometrial tissues and endometrial cancer tissues. To further validate ALYREF expression, RNA sequencing data from The Cancer Genome Atlas Uterine Corpus Endometrial Carcinoma (TCGA‐UCEC) cohort were integrated with normal uterine tissue data from the Genotype‐Tissue Expression (GTEx) database for differential expression analysis. In addition, paired tumor and adjacent normal tissue samples from the TCGA‐UCEC cohort were analyzed to compare ALYREF expression between matched tissues. Protein expression of ALYREF in endometrial cancer was further evaluated using immunohistochemical staining images obtained from The Human Protein Atlas (HPA) database (https://www.proteinatlas.org/).

The prognostic significance of ALYREF was assessed using the KM Plotter database (https://kmplot.com/analysis/). Overall survival (OS) and relapse‐free survival (RFS) were analyzed by stratifying patients into high‐ and low‐expression groups using the Auto select best cutoff option provided by the platform, which automatically determines the optimal cutoff value for survival analysis. Kaplan–Meier survival curves were generated automatically, and differences between groups were compared using the log‐rank test.

To investigate the association between ALYREF expression and clinicopathological characteristics, the TCGA‐UCEC cohort was further analyzed according to histologic grade, histological type, depth of myometrial invasion, clinical stage, and age. Comparisons among multiple groups were performed using the Kruskal–Wallis test followed by Dunn's multiple comparison test, whereas comparisons between two groups were conducted using the Wilcoxon rank‐sum test. In addition, Cox proportional hazards regression analyses were performed to evaluate the prognostic significance of ALYREF and XRCC6. Variables with statistical significance in the univariate analysis were subsequently included in the multivariate Cox regression model to identify independent prognostic factors for overall survival. Hazard ratios (HRs) and 95% confidence intervals (CIs) were calculated. All statistical tests were two‐sided, and a *p* value < 0.05 was considered statistically significant.

### 5.2 Clinical Specimens

Paraffin‐embedded tissue samples were obtained from 50 EC tissues and 20 normal endometrial tissues collected from patients who underwent treatment at the Second Hospital of Jilin University between January 2017 and December 2022. Paraffin sections were prepared at a thickness of 4 µm using standard protocols. This study was approved by the Ethics Committee of the Second Hospital of Jilin University. Written informed consent was obtained from all patients before sample collection.

### 5.3 Immunohistochemistry (IHC)

Paraffin sections were sequentially subjected to deparaffinization, rehydration, and antigen retrieval. After blocking with 5% BSA, the sections were incubated with the primary antibody. The primary antibody was incubated overnight at 4°C, followed by incubation with a biotin‐labeled secondary antibody at room temperature for 1 h. DAB solution was used for visualization of the immunoreactivity. The antibody used was anti‐ALYREF (1:500, ab202894, Abcam) and anti‐XRCC6 (1:500, A0883, ABclonal). All IHC slides were independently evaluated by two experienced pathologists in a blinded manner, without prior knowledge of the patients' clinicopathological characteristics or clinical outcomes, and any discrepant cases were jointly reviewed under a multi‐head microscope to reach a consensus. Protein expression and its clinical relevance were quantified using two targeted analytical methods according to the specific evaluation objectives. To quantify the basic expression level and differential expression of ALYREF, digital optical density analysis was performed using ImageJ software (NIH, Bethesda, MD, USA); five random high‐power fields (× 400) per slice were captured to measure the Integrated Density (IntDen) and Area, with the relative expression level defined as IntDen/ Area. To evaluate the correlation between ALYREF and XRCC6 expression, the H‐score method was applied. The H‐score was calculated using the formula: H‐score = (% of weak cells × 1) + (% of moderate cells × 2) + (% of strong cells × 3), yielding a continuous score ranging from 0 to 300, where staining intensity was graded as 0 (negative), 1 (weak), 2 (moderate), or 3 (strong).

### 5.4 RNA Extraction and Quantitative Real‐Time PCR (RT‐qPCR)

Total RNA was extracted from cells using an RNA Extraction Kit (Vazyme, China). Cytoplasmic and nuclear RNA were extracted separately using the Cytoplasmic and Nuclear RNA Purification Kit (Norgen Biotek, Canada). Reverse transcription of RNA into cDNA was performed using the PrimeScript RT Master Mix Kit (TaKaRa, Japan). Quantitative PCR (qPCR) was performed using the ChamQ Universal SYBR qPCR Master Mix (Vazyme, China). The sequences of RT‐qPCR primers are provided in Table S2.

### 5.5 Western Blotting

Cells were lysed on ice for 30 min using RIPA lysis buffer supplemented with 1% PMSF (Beyotime, China). The lysates were centrifuged at 13 000 rpm for 10 min at 4°C. The supernatant was transferred to a new EP tube, and 15 µL aliquots were used to measure protein concentration using the BCA Protein Assay. Subsequently, 6 × loading buffer (TransGen Biotech, China) was added, and the samples were heated at 100°C for 10 min to denature the proteins. Protein samples were separated by SDS‐PAGE and transferred onto PVDF membranes. The membranes were blocked with 5% non‐fat milk and then incubated sequentially with primary and secondary antibodies. The antibody information is provided in Table S3.

### 5.6 Immunofluorescence

A total of 2 × 10^5^ cells were seeded onto coverslips in 12‐well plates and cultured overnight. After washing with PBS, the cells were blocked with 500 µL of blocking solution at room temperature for 1 h. Diluted primary antibodies were then added and incubated overnight at 4°C. Following removal of the primary antibodies, the cells were washed three times with PBS for 10 min each time. Under light‐protected conditions, secondary antibodies and Hoechst were added and incubated at room temperature for 1–2 h, followed by two washes with PBS, each lasting 5 min. The coverslips were inverted onto microscope slides containing an anti‐fade mounting medium, sealed with cover slip sealant, stored in the dark, and subsequently observed and imaged using a fluorescence microscope. The antibody information is provided in Table S3.

### 5.7 Cell Culture

The EC cell lines HEC‐1A and Ishikawa were purchased from the Institute of Basic Medical Sciences for Chinese Academy of Medical Sciences (Beijing, China). KLE cells were obtained from Fuheng Biology (Shanghai, China). All cell lines were authenticated by short tandem repeat (STR) profiling and tested for mycoplasma contamination prior to use. HEC‐1A cells were maintained in McCoy's 5A medium, Ishikawa cells in high‐glucose DMEM, and KLE cells in MEM medium. Complete media were prepared by supplementing the respective basal media with 10% fetal bovine serum (FBS) and 1% penicillin‐streptomycin (100 ×). All cells were cultured at 37°C in a humidified atmosphere containing 5% CO_2_.

### 5.8 Lentiviral Infection and siRNA

A lentiviral vector system carrying a FLAG tag and a puromycin resistance gene was provided by HanHeng Biotechnology (Shanghai, China). Cells were first seeded in 12‐well plates and then infected with either a lentivirus carrying the target gene or an empty vector control. After 48 h post‐infection, stably transduced cell lines were selected using puromycin. The efficiency of gene knockdown or overexpression was verified by RT‐qPCR and Western blotting analysis. SiRNAs were synthesized by GenePharma (Shanghai, China). For gene silencing, cells were transfected with siRNAs using the GP‐Transfect‐Mate Transfection Reagent from GenePharma (Shanghai, China). The targeted sequences of shRNAs or siRNAs are provided in Table S4.

### 5.9 Cell Proliferation Assay

For the colony formation assay, cells in the logarithmic growth phase were digested into a single‐cell suspension and counted. A total of 1000 cells per well were seeded into 6‐well plates according to the experimental groups. The cells were cultured for 7–14 days, with the medium replaced every 3 days. When colonies contained more than 50 cells, they were washed with PBS and fixed in 4% paraformaldehyde for 20 min. After fixation, colonies were washed with PBS, stained with 5% crystal violet solution, and washed three times with PBS. Plates were air‐dried, photographed, and analyzed.

For the Cell Counting Kit‐8 (CCK‐8) assay, cells in the logarithmic growth phase were digested into a single‐cell suspension and counted. A total of 2,000 cells per well were seeded in 96‐well plates according to the experimental groups. After cell attachment, the medium was replaced at 0, 24, 48, 72, and 96 h with 100 µL of fresh medium containing 10% CCK‐8 reagent in per well. The cells were incubated for 2 h at 37°C in the dark. Absorbance at 450 nm was measured, and the optical density (OD) values were recorded and analyzed.

For the 5‐Ethynyl‐2′‐deoxyuridine (EdU) incorporation assay, cells in the logarithmic growth phase were digested into a single‐cell suspension and counted. A total of 2 × 10^5^ cells per well were seeded on coverslips placed in 12‐well plates and cultured overnight. Cells were labeled with 10 µM EdU working solution (HEC‐1A: 4 h; Ishikawa: 2 h) at 37°C. After fixation with 4% paraformaldehyde for 15 min and permeabilization with 0.3% Triton X‐100 for 10 min, the Click reaction was performed in the dark for 30 min. Nuclei were stained with Hoechst 33342 for 10 min. After washing with PBS, coverslips were mounted using anti‐fade mounting medium, and images were captured with a fluorescence microscope.

### 5.10 Cell Migration Assay and Invasion Assay

For the wound healing assay, EC cells in the logarithmic growth phase were seeded into 6‐well plates. When cell confluence exceeded 90%, a straight‐line scratch was created using a pipette tip. After washing with PBS, the medium was replaced with medium containing 1% FBS. Images of the wound area were captured at 0 and 48 h. Wound width was measured using ImageJ software, and the migration rate was calculated as: migration rate = [(Area at 0 h − Area at 48 h) / Area at 0 h] × 100%.

For Transwell migration and invasion assays, cells in good condition were serum‐starved for 24 h. For invasion assays, Matrigel was thawed at 4°C overnight, and pipette tips were pre‐cooled in advance. Matrigel was diluted on ice to 1 mg mL^−1^, and 60 µL was carefully added to the upper chamber of the Transwell insert and incubated at 37°C for 1–3 h to solidify, followed by pre‐hydration with serum‐free medium for 30 min (this step was omitted for migration assays). In the lower chamber, 500 µL of complete medium containing 10% FBS was added, while 100 µL of serum‐free cell suspension (2 × 10^5^ cells mL^−1^) was seeded into the upper chamber. Cells were incubated at 37°C with 5% CO_2_ for 24–48 h. After incubation, the inserts were washed with PBS, fixed with 4% paraformaldehyde for 30 min, and stained with crystal violet for 15 min. After rinsing with water and air‐drying, 3–5 random fields per membrane were imaged under a microscope, and cell numbers were quantified using Image J software.

### 5.11 Cell Cycle Analysis

The cell cycle distribution was analyzed by propidium iodide (PI) staining combined with flow cytometry. Briefly, 1 × 10^6^ cells were harvested, washed with PBS, and fixed overnight in 70% pre‐cooled ethanol. The following day, cells were washed with PBS to remove ethanol and then stained with 500 µL of PI solution in the dark for 30 min. The samples were then analyzed immediately using flow cytometry. DNA content distribution was used to determine the cell cycle phases.

### 5.12 Cell Apoptosis Analysis

Apoptosis was assessed using Annexin V‐APC/7‐AAD dual staining followed by flow cytometry. Briefly, 5 × 10^5^ cells were harvested, washed with PBS, and resuspended in 100 µL of binding buffer. Subsequently, 2.5 µL of Annexin V‐APC and 2.5 µL of 7‐AAD were added, and the mixture was incubated in the dark for 30 min. After 400 µL of binding buffer was added, the samples were immediately analyzed using flow cytometry.

### 5.13 Tumor Xenograft Model

Female BALB/c nude mice (4–5 weeks old) were purchased from Sibeifu Animal Technology (Beijing, China) and maintained under specific pathogen‐free (SPF) barrier conditions. A total of 5 × 10^6^ cells suspended in 100 µL of medium were subcutaneously injected into the right flank of each mouse. The health status and tumor growth were monitored daily. When the tumor volume reached approximately 150 mm^3^, the mice were euthanized, and tumors were harvested for weight measurement and imaging. All animal experiments were approved by the Laboratory Animal Welfare and Ethics Committee of the School of Public Health, Jilin University.

### 5.14 RNA Sequencing (RNA‐seq)

RNA‐seq was conducted by BGI Genomics Co., Ltd (Shenzhen, China). Total RNA was extracted using the TRIzol reagent. RNA purity (OD_260/280_ = 1.8–2.2) was assessed using a NanoDrop spectrophotometer, and RNA integrity (RIN > 7.0) was evaluated with an Agilent 2100 Bioanalyzer. Qualified RNA samples were used for mRNA library preparation via poly(A) enrichment or rRNA depletion. Following RNA fragmentation, reverse transcription, end repair, adapter ligation, and PCR amplification, cDNA libraries were constructed. Paired‐end 150 bp sequencing (PE150) was performed on the BGISEQ‐500. Raw data were processed through quality control (FastQC), decontamination, alignment (using HISAT2 or STAR), and quantification (using FeatureCounts). Differentially expressed genes (DEGs) were identified using DESeq2 or edgeR and analyzed for Gene Ontology (GO) and Kyoto Encyclopedia of Genes and Genomes (KEGG) enrichment. All sequencing data have been deposited in a publicly accessible database.

### 5.15 RNA Immunoprecipitation Sequencing (RIP‐seq)

RIP was conducted using the Magna RIP Kit (Millipore, Germany) according to the manufacturer's instructions. Briefly, HEC‐1A cells were lysed with RIP lysis buffer supplemented with protease and RNase inhibitors to prepare cell lysates. The supernatants were incubated overnight at 4°C with magnetic beads conjugated to anti‐ALYREF antibodies (ab202894, Abcam) or anti‐NSUN2 antibodies(Abclonal, A24132). The RNA‐protein complexes were subsequently washed, eluted, and purified. Purified RNA was subjected to strand‐specific library preparation and sequencing, which was performed by Guangzhou Epibiotek Co., Ltd (China). Library quality was assessed using a Bioptic Qsep100 system. Sequencing was performed on the Illumina platform with paired‐end 150 bp (PE150) reads. Bioinformatic analysis included adapter trimming, quality control, genome alignment, quantification, and functional annotation. All sequencing data have been deposited in a publicly accessible database.

### 5.16 5‐Methylcytosine Bisulfite Sequencing (m^5^C‐BS‐seq)

m^5^C‐BS‐seq was conducted by Shanghai Cloud‐Seq Biotech Co., Ltd (China). rRNA was removed from total RNA using the GenSeq rRNA Depletion Kit. Bisulfite conversion and purification were performed using the EZ RNA Methylation Kit. Libraries were prepared using a GenSeq Low Input RNA Library Preparation Kit. Library quality was assessed using an Agilent 2100 Bioanalyzer. High‐throughput sequencing was then performed on the Illumina platform. For bioinformatic analysis, quality control was performed using Q30 metrics, FastQC, and FastQ Screen. Adapter sequences and low‐quality reads were trimmed with Cutadapt. Clean reads were aligned to the reference genome using meRanGs, and genome‐wide m^5^C sites were identified using meRanCall or meRanCompare. Functional annotation and enrichment analysis of m^5^C sites on mRNA were subsequently performed. All sequencing data have been deposited in a publicly accessible database.

### 5.17 RNA Stability Assay

Cells in the logarithmic growth phase from each group were treated with 5 µg mL^−1^ Actinomycin D, and samples were collected at 0, 4, 8, and 12 h post‐treatment. Total RNA was extracted using the TRIzol reagent, and the expression of the target gene was quantified by RT‐qPCR.

### 5.18 Dual‐Luciferase Assay

Cells were seeded into 24‐well plates and transfected with 500 ng of either wild‐type (WT) or mutant (MUT) *XRCC6*‐CDS firefly luciferase reporter plasmids, along with 100 ng of the pRL‐SV40 Renilla luciferase reporter plasmid, using GP‐transfect‐Mate reagent (GenePharma, China). After 48 h, luciferase activity was measured using a Dual‐Luciferase Reporter Assay Kit (Beyotime, China). Renilla luciferase activity was used for normalization.

### 5.19 TCF/LEF‐Dependent Luciferase Reporter Assay

To evaluate β‐catenin TCF‐mediated transcriptional activity, EC cells seeded in 24‐well plates were co‐transfected with TOPflash or mutant FOPflash reporter plasmids together with the pRL‐TK internal control plasmid using Lipofectamine 3000 (Invitrogen). After 48 h, Firefly and Renilla luciferase activities were quantified using the Dual‐Luciferase Reporter Assay System (Promega). Firefly luciferase activity was normalized to Renilla luciferase activity, and the relative β‐catenin/TCF transcriptional activation was calculated as the TOPflash‐to‐FOPflash ratio.

### 5.20 Co‐Immunoprecipitation (Co‐IP)

HEC‐1A cells were lysed in cold NP‐40 lysis buffer containing a protease inhibitor cocktail (Roche), followed by centrifugation to collect clarified protein lysates. Equal amounts of protein were incubated overnight at 4°C with an anti‐XRCC6 antibody or control rabbit IgG, and immunocomplexes were captured using Protein A/G magnetic beads at 4°C for 2 h. After thorough washing with NP‐40 buffer, proteins were eluted with SDS sample buffer, resolved by SDS‐PAGE, and analyzed by Western blotting. Co‐precipitated β‐catenin and immunoprecipitated XRCC6 were detected using their corresponding primary antibodies.

### 5.21 Statistical Analysis

Statistical analyses were performed using GraphPad Prism version 9 software. Data are presented as the mean ± standard error of the mean (SEM) from at least three independent biological replicates. Differences between two groups were assessed using Student's t‐test, while comparisons among three or more groups were evaluated using one‐way ANOVA. ***p < 0.001, **p < 0.01, and *p < 0.05 were considered statistically significant, and *p* > 0.05 was considered not significant (ns). The correlation between ALYREF and XRCC6 expression levels in tissue samples was evaluated using Spearman's rank correlation analysis.

## Author Contributions

**Jie Guo**, **Linlin Hao**, and **Jian Zhang** contributed to the experimental design. **Linlin Hao** and **Jian Zhang** conducted data analysis and prepared the manuscript. **Linlin Hao** and **Jian Zhang** carried out most of the experiments. **Qi Qian**, **Min Jiang**, and **Yu Liu** were responsible for the collection of clinical samples and related data. **Jie Guo**, **Qi Qian**, **Yan Geng**, **Nan Cui**, and **Qi Guo** critically reviewed and revised the manuscript. All authors have read and approved the final version of the manuscript.

## Conflicts of Interest

The authors declare no conflicts of interest.

## Supporting information




**Supporting File**: advs77548‐sup‐0001‐SuppMat.docx.

## Data Availability

RNA‐seq (GSE304342), RIP‐seq (GSE304341), and m^5^C‐BS‐seq (GSE304340) datasets generated in this study are available in the Gene Expression Omnibus (GEO).
