# Supplementary material for: m5C Reader ALYREF Promotes the Progression of Endometrial Cancer by Activating the XRCC6‐Mediated Wnt/β‐catenin Signaling Pathway
Source: Adv Sci (Weinh). 2026 Sep 27:e77548. Online ahead of print. doi: 10.1002/advs.77548 (PMC13616237; doi:10.1002/advs.77548)
Supplement: Supplementary file 1 [file ADVS-9999-e77548-s001.docx]

Supporting Information for

**m^5^C Reader ALYREF Promotes the Progression of Endometrial Cancer by Activating the XRCC6-Mediated Wnt/β-catenin Signaling Pathway**

Linlin Hao^1,2,3,4#^, Jian Zhang^5#^, Qi Qian^1^, Min Jiang^1^, Yu Liu^1^, Yan Geng^1^, Nan Cui^1^, Qi Guo^1^, Jie Guo^1*^

Table of contents

Supplementary figures (1-9)

Supplementary tables (1-4)

Supplementary Data (Full length western blotting Images)

**Figure S1 Association between ALYREF expression and clinicopathological characteristics in the TCGA-UCEC cohort.** (A–C) ALYREF expression levels were analyzed according to clinicopathological parameters in the TCGA-UCEC cohort, including (A) clinical stage, (B) histologic grade, and (C) histological type. ALYREF expression is presented as log₂ (TPM + 1). Statistical significance among multiple groups was determined using the Kruskal–Wallis test followed by Dunn’s multiple comparisons test. **P* < 0.05, ***P* < 0.01, ****P* < 0.001.

**Figure S2. ALYREF overexpression promotes proliferation of KLE cells.** (A) RT-qPCR analysis confirming *ALYREF* mRNA overexpression in KLE cells transduced with empty vector (Vector) or ALYREF overexpression lentiviral vector (OE-ALYREF). (B) Western blot analysis confirming ALYREF protein overexpression in KLE cells transduced with *ALYREF* overexpression lentiviral vector. (C) Cell proliferation was evaluated using CCK-8 assay in KLE cells with ALYREF overexpression. Cell viability was measured at the indicated time points. (D, E) Representative images (D) and quantification (E) of colony formation assays showing that ALYREF overexpression increased the clonogenic capacity of KLE cells. (F, G) Representative EdU staining images (F) and quantitative analysis (G) demonstrating that ALYREF overexpression enhanced DNA synthesis and proliferation of KLE cells. EdU-positive cells are shown in red, nuclei are stained with Hoechst (blue), and merged images are shown. **P* < 0.05, ***P* < 0.01, ****P* < 0.001. scale bar = 100 μm.

**Figure S3 Functional effects of ALYREF knockdown on migration, invasion, cell cycle progression, and apoptosis in Ishikawa cells.** (A) Wound healing assay assessing the effect of ALYREF knockdown on the migration of Ishikawa cells. (B) Transwell assays evaluating the migration and invasion abilities of Ishikawa cells after ALYREF knockdown, with corresponding quantification of migrated and invaded cells. (C) Cell cycle analysis by PI staining and flow cytometry after ALYREF knockdown in Ishikawa cells, with quantification of cells in G0/G1, S, and G2/M phases. (D) Apoptosis analysis using Annexin V/7-AAD staining and flow cytometry after ALYREF knockdown in Ishikawa cells, with quantification of apoptotic cells. (E) Wound healing assay assessing the effect of ALYREF overexpression (OE-ALYREF) on the migration of KLE cells. (F) Transwell assays evaluating the migration and invasion abilities of KLE cells after ALYREF overexpression, with corresponding quantification. (G) Cell cycle analysis and quantification after ALYREF overexpression in KLE cells. (H) Apoptosis analysis and quantification after ALYREF overexpression in KLE cells. **P* < 0.05, ***P* < 0.01, ****P* < 0.001; ns: non-significant; scale bar = 100 μm.

**Figure S4 Regulation of XRCC6 by ALYREF and their expression correlation in endometrial cancer tissues.** (A) Western blot analysis of XRCC6 protein level in Ishikawa cells after *ALYREF* knockdown compared with the negative control. (B, C) Immunofluorescence (IF) staining showing the expression and localization of XRCC6 (red) in (B) HEC-1A cells and (C) Ishikawa cells following *ALYREF* knockdown. Nuclei were counterstained with Hoechst (blue). (D, E) Representative immunohistochemistry (IHC) images of ALYREF and XRCC6 expression in (D) normal endometrial tissues and (E) endometrial cancer tissues. Enlarged views of the boxed areas are shown on the right. (F) Spearman’s correlation analysis between the IHC H-score of ALYREF and XRCC6 in endometrial cancer tissues (*n* = 50, Spearman *r* = 0.82, *P* < 0.001). Scale bars = 100 $\mu$m.

**Figure S5 XRCC6 overexpression rescues the inhibitory effects of *ALYREF* knockdown on proliferation, migration, invasion, cell cycle progression, and apoptosis in Ishikawa cells.** (A) CCK-8 assay demonstrating restoration of proliferation upon *XRCC6* overexpression in *ALYREF*-knockdown Ishikawa cells. (B) Colony formation assay assessing whether *XRCC6* overexpression rescues *ALYREF* knockdown-induced suppression of proliferation in Ishikawa cells, with (C) quantification of colony numbers. (D) EdU incorporation assay assessing the effect of XRCC6 overexpression on cell proliferation under *ALYREF* knockdown, with (E) quantification of the percentage of EdU-positive cells. (F) Wound healing assay and quantitative analysis of migration rates assessing the impact of XRCC6 overexpression on cell migration in *ALYREF*-knockdown Ishikawa cells. (G) Transwell assays and corresponding quantification of migrated and invaded cells in *ALYREF*-knockdown Ishikawa cells with or without *XRCC6* overexpression. (H) Cell cycle analysis by PI staining and flow cytometry, with quantification of cells across different phases. (I) Apoptosis analysis using Annexin V/7-AAD staining and flow cytometry, with quantification of apoptotic cells. **P* < 0.05, ***P* < 0.01, ****P* < 0.001; ns: not significant; scale bars = 100 μm.

**Figure S6. ALYREF associates with *XRCC6* mRNA in HEC-1A cells.** RIP-qPCR using anti-ALYREF antibody showed significant enrichment of *XRCC6* mRNA compared with IgG control. ****P* < 0.001

**Figure S7. Validation of methyltransferase knockdown efficiency, m^5^C site mapping, and schematic of mutant constructs for XRCC6.** (A, B) RT-qPCR validation of the knockdown efficiency of (A) *NSUN2* and (B) *NSUN6* in HEC-1A and Ishikawa cells. The relative expression levels of *NSUN2* and *NSUN6* mRNA were measured relative to the shNC control group. (C) IGV (Integrative Genomics Viewer) track visualization of m^5^C modification sites on *XRCC6* mRNA derived from the m^5^C-BS-seq dataset, showing high-confidence methylation peaks within the target locus (magnified in the dashed box). (D) In silico prediction of the m^5^C modification site on *XRCC6* mRNA (chr22:41637761) retrieved from the RMVar 2.0 database. (E) Schematic illustration of the luciferase reporter constructs containing either the wild-type *XRCC6* sequence (*XRCC6*-WT) or the mutated *XRCC6* sequence (XRCC6-MUT), in which the specific cytosines (CC) at the predicted m^5^C site were substituted with adenines (AA). (F) Schematic illustration of WT, single-site mutant (Mut1 and Mut2), and double-site mutant (Double Mut) *XRCC6* mRNA constructs fused with 3×FLAG. The two m^5^C consensus motifs (CGCGCA) within the XRCC6 coding sequence (CDS) are indicated.

**Figure S8. ALYREF promotes canonical Wnt/β-catenin signaling and downstream target transcription via XRCC6 in endometrial cancer cells.** (A–C) Evaluation of β-catenin nuclear translocation and downstream protein expression in Ishikawa cells following *ALYREF* knockdown. (A) Representative immunofluorescence (IF) images showing the subcellular localization of β-catenin (red). Nuclei were counterstained with Hoechst (blue). Cropped and magnified views of the indicated areas are presented on the right. (B) Statistical quantification of the percentage of cells presenting nuclear β-catenin localization. (C) Western blot analysis of total β-catenin, nuclear β-catenin, and c-Myc expression levels. β-actin and Histone H3 (H3) were utilized as loading controls for the total/cytoplasmic and nuclear fractions, respectively. (D–F) Rescue effects of XRCC6 overexpression (OE-XRCC6) on β-catenin signaling in *ALYREF*-knockdown Ishikawa cells. (D) Representative IF images, (E) corresponding statistical quantification of nuclear β-catenin localization, and (F) Western blot analysis of flag-XRCC6, total β-catenin, nuclear β-catenin, and c-Myc levels under the specified transfection conditions. (G) Co-immunoprecipitation (Co-IP) assay in HEC-1A cells using an anti-XRCC6 antibody or control IgG, followed by Western blot analysis with anti-β-catenin and anti-XRCC6 antibodies to assess their endogenous physical interaction. (H–K) RT-qPCR validation of the rescue effect of *XRCC6* overexpression on the mRNA levels of canonical Wnt target genes after *ALYREF* knockdown, including (H) AXIN2 in HEC-1A cells, (I) AXIN2 in Ishikawa cells, (J) CCND1 in HEC-1A cells, and (K) CCND1 in Ishikawa cells. **P* < 0.05, ***P* < 0.01, ****P* < 0.001; scale bars = 100 μm.

**Figure S9**. **XRCC6-mediated proliferative rescue relies on the canonical Wnt/β-catenin signaling pathway.** (A, B) CCK-8 assays showing the proliferation of HEC-1A (A) and Ishikawa (B) cells transfected with shNC + vector, sh*ALYREF* + vector, sh*ALYREF* + *XRCC6*, or sh*ALYREF* + *XRCC6* treated with the Wnt inhibitor XAV-939. (C, D) Representative images (left) and quantification (right) of colony formation assays in HEC-1A (C) and Ishikawa (D) cells under the indicated treatment conditions. **P* < 0.05, ***P* < 0.01, ****P* < 0.001.

Table S1. Univariate and multivariate Cox regression analyses of clinicopathological characteristics and ALYREF expression for overall survival in patients with TCGA-UCEC

| **Characteristics** | **Total**  **(N)** | **Univariate analysis** | |  | **Multivariate analysis** | |
| --- | --- | --- | --- | --- | --- | --- |
|  |  | **Hazard ratio**  **(95% CI)** | **P value** |  | **Hazard ratio**  **(95% CI)** | **P value** |
| **Age** | 551 |  |  |  |  |  |
| <= 60 | 207 | Reference |  |  | Reference |  |
| > 60 | 344 | 1.850 (1.162 - 2.944) | **0.009** |  | 1.561 (0.947 - 2.575) | 0.081 |
| **Clinical stage** | 553 |  |  |  |  |  |
| Stage I | 342 | Reference |  |  | Reference |  |
| Stage II | 52 | 1.738 (0.833 - 3.626) | 0.140 |  | 1.469 (0.696 - 3.101) | 0.313 |
| Stage III | 130 | 3.084 (1.911 - 4.977) | **< 0.001** |  | 2.512 (1.496 - 4.219) | **< 0.001** |
| Stage IV | 29 | 8.082 (4.497 - 14.524) | **< 0.001** |  | 5.689 (3.009 - 10.756) | **< 0.001** |
| **Histologic grade** | 542 |  |  |  |  |  |
| G1 | 99 | Reference |  |  | Reference |  |
| G2 | 121 | 7.111 (1.616 - 31.297) | **0.009** |  | 6.056 (1.370 - 26.775) | **0.018** |
| G3 | 322 | 13.303 (3.262 - 54.242) | **< 0.001** |  | 7.799 (1.858 - 32.733) | **0.005** |
| **Histological type** | 553 |  |  |  |  |  |
| Endometrioid | 411 | Reference |  |  | Reference |  |
| Mixed | 24 | 2.428 (1.040 - 5.672) | **0.040** |  | 1.643 (0.678 - 3.984) | 0.272 |
| Serous | 118 | 2.674 (1.744 - 4.100) | **< 0.001** |  | 1.123 (0.670 - 1.882) | 0.659 |
| **ALYREF** | 553 |  |  |  |  |  |
| Low | 277 | Reference |  |  | Reference |  |
| High | 276 | 5.362 (3.123 - 8.052) | **< 0.001** |  | 3.765(2.562-6.871) | **0.031** |
| **XRCC6** | 553 |  |  |  |  |  |
| Low | 277 | Reference |  |  | NA | NA |
| High | 276 | 1.160 (0.770 - 1.746) | 0.479 |  | NA | NA |

Abbreviations: HR, hazard ratio; CI, confidence interval; NA, not applicable. XRCC6 was not included in the multivariate Cox regression analysis because it was not statistically significant in the univariate analysis.

Table S2 Sequences of primers used for qRT-PCR in this study.

| Name | Sequence（5' -3 '） |
| --- | --- |
| *ALYRE*F forward | TGCCACCTCTGTTTACGCTC |
| *ALYREF* reverse | TCTGGTCGCAGCTTAGGAAC |
| *HYOU1* forward | GAGGAGGCGAGTCTGTTGG |
| *HYOU1* reverse | GCACTCCAGGTTTGACAATGG |
| *CCDC71* forward | CTCGTGCCCCTAACCCAAC |
| *CCDC71* reverse | CTGCCAGATAGCGGCATGG |
| *FDXR* forward | CAGCATTGGGTATAAGAGCCG |
| *FDXR* reverse | GGCCTGGCACATCCATAACC |
| *XRCC6* forward | TTGCTTCTGCCTAGCGATACC |
| *XRCC6* reverse | AAACCTGGATCATCAAACCGTT |
| *SHMT2* forward | CCCTTCTGCAACCTCACGAC |
| *SHMT2* reverse | TGAGCTTATAGGGCATAGACTCG |
| *MCM7* forward | ACTCTCAGAAACCTACCTGGAAG |
| *MCM7* reverse | CAGCTTTTCGTAGAAATCCTCCT |
| *IL17RC* forward | GATGGTGACAACGTGCATCTG |
| *IL17RC* reverse | CAAGGTAATGATCTGCGGTCC |
| *PYGB* forward | ACCCCAAGCGCATTTATTATCTT |
| *PYGB* reverse | GGCTTCATCGCAGGCATTC |
| *MYDGF* forward | ACACAGTTCAAGGCAGAGG |
| *MYDGF* reverse | TCAGTTTTCAGAGGGACATCAC |
| *APEH* forward | ATGTCGAGAGTGGCAACATCT |
| *APEH* reverse | GCGATTGGTGCAAAAGCGG |
| *NSUN2* forward | GGTCAACCATGATGCCTCCA |
| *NSUN2* reverse | AATCCGCAGCTGTAAGCCAT |
| *NSUN6* forward | TTGCAACGTGAGTGGCATTG |
| *NSUN6* reverse | GCTTTCTCAAGCCTAGCCGA |
| *GAPDH* forward | GGAGTCCACTGGCGTCTTCA |
| *GAPDH* reverse | GTCATGAGTCCTTCCACGATACC |

Table S2 Sequences of primers used for qRT-PCR in this study (Continued).

| Name | Sequence（5' -3 '） |
| --- | --- |
| AXIN2 forward | AGGCCCTGCTGTAAAAGAGAG |
| *AXIN2* reverse | AGTTCCTCTCAGCAATCGGC |
| *CCND1* forward | ATCAAGTGTGACCCGGACTG |
| *CCND1* reverse | CTTGGGGTCCATGTTCTGCT |

Table S3 Antibody used in this study

| Antibody | company | Catalogue number | Concentration |
| --- | --- | --- | --- |
| Anti_ALYREF | Abcam | ab202894 | 1:5000(WB), 1:2000(IF) |
| Anti_XRCC6 | ABclonal | A0883 | 1:2500(WB), 1:1000(IF) |
| Anti_β-catenin | Proteintech | 51057-2-AP | 1:2000(WB), 1:500(IF) |
| Anti_c-Myc | Proteintech | 10828-1-AP | 1:1000(WB) |
| Anti_β-actin | ABclonal | AC028 | 1:2500(WB) |
| Anti_H3 | Proteintech | 17168-1-AP | 1:2000(WB) |

Table S4. ShRNA and siRNA used for silencing target genes.

| Target transcript | Sequence |
| --- | --- |
| shNC: | 5′‐GCATAAACCCGCCACTCATCT‐3′ |
| sh*ALYREF*#1： | 5′‐CGTGGAGACAGGTGGGAAACT‐3′ |
| sh*ALYREF*#2： | 5′‐ GGAGTCTCAGACGCCGATATT‐3′ |
| sh*NSUN2*#1： | 5′‐ GAGCGATGCCTTAGGATATTA‐3′ |
| sh*NSUN2*#2： | 5′‐ TGCAGTGTCCCATCGTCTTAT‐3′ |
| sh*NSUN6*#1：  shNSUN6#2 | 5′‐ GCTGGAGATGTTATTTCTGTA‐3′  5′‐ GAGAAGTTATAGCACTGGATA‐3′ |
| siCtrl | 5′-UUCUCCGAACGUGUCACGUTT-3′ |
| si*ALYREF* | 5′-GGAGUCUCAGACGCCGAUAUUTT -3′ |

**Full length western blotting Images**

**1.Blots for Figure 2B:**

Full-length blots showing endogenous ALYREF protein levels (left panel, expected molecular weight: 27 kDa) and the corresponding loading control β-actin (right panel, expected molecular weight: 42 kDa) across HEC-1A, Ishikawa, and KLE cell lines. Protein molecular weight markers (kDa) are indicated on the right side of each blot.

**2.Blots for Figure 2D (HEC-1A)**

Full-length blots showing ALYREF knockdown efficiency in HEC-1A cells transfected with shNC, sh*ALYREF*#1, or sh*ALYREF*#2 vectors (left panel, 27 kDa) and the matching β-actin control (right panel, 42 kDa). Molecular weight markers (kDa) are explicitly labeled on the right.

**3. Blots for Figure 2D (Ishikawa)**

Full-length blots showing ALYREF knockdown efficiency in Ishikawa cells transfected with shNC, sh*ALYREF*#1, or sh*ALYREF*#2 vectors (left panel, 27 kDa) and the matching β-actin control (right panel, 42 kDa). Molecular weight markers (kDa) are explicitly labeled on the right.

**4.Blots for Figure S2B (KLE)**

Full-length blots showing ALYREF protein expression levels in Ishikawa cells infected with different doses of ALYREF-overexpressing lentivirus (Vector, OE-ALYREF (3 μ), or OE-ALYREF (5 μ)) (left panel, expected molecular weight: 27 kDa) and the corresponding loading control β-actin (right panel, expected molecular weight: 42 kDa). Red dashed boxes indicate the cropped regions presented in the main figure. Protein molecular weight markers (kDa) are indicated on the right side of each blot.

**5.Blots for Figure 4K (HEC-1A)**

HEC-1A

Full-length blots showing XRCC6 protein expression levels following ALYREF knockdown (shNC, sh*ALYREF*#1, or sh*ALYREF*#2 vectors) in HEC-1A cells (left panel, expected molecular weight: 70 kDa) and the corresponding loading control β-actin (right panel, expected molecular weight: 42 kDa). Protein molecular weight markers (kDa) are indicated on the right side of each blot.

**6.Blots for Figure S4A (Ishikawa)**

Full-length blots showing XRCC6 protein expression levels following ALYREF knockdown (shNC, sh*ALYREF*#1, or sh*ALYREF*#2 vectors) in Ishikawa cells (left panel, expected molecular weight: 70 kDa) and the corresponding loading control β-actin (right panel, expected molecular weight: 42 kDa). Protein molecular weight markers (kDa) are indicated on the right side of each blot.

**7. Blots for Figure 8C (HEC-1A)**

Full-length blots showing protein expression levels in HEC-1A cells following ALYREF knockdown (shNC, sh*ALYREF*#1, or sh*ALYREF*#2 vectors). Top panels: Total β-catenin levels (left, expected molecular weight: 92 kDa) and its corresponding loading control β-actin (right, 42 kDa).Middle panels: Nuclear β-catenin levels (left, expected molecular weight: 92 kDa) and its corresponding nuclear loading control Histone H3 (right, H3, 16 kDa).Bottom panels: c-Myc levels (left, expected molecular weight: 62 kDa) and its corresponding loading control β-actin (right, 42 kDa).Protein molecular weight markers (kDa) are indicated on the sides of each blot.

**8. Blots for Figure S8C (Ishikawa)**

Full-length blots showing protein expression levels in Ishikawa cells following ALYREF knockdown (shNC, sh*ALYREF*#1, or sh*ALYREF*#2 vectors). Top panels: Total β-catenin levels (left, expected molecular weight: 92 kDa) and its corresponding loading control β-actin (right, 42 kDa).Middle panels: Nuclear β-catenin levels (left, expected molecular weight: 92 kDa) and its corresponding nuclear loading control Histone H3 (right, H3, 16 kDa).Bottom panels: c-Myc levels (left, expected molecular weight: 62 kDa) and its corresponding loading control β-actin (right, 42 kDa).Protein molecular weight markers (kDa) are indicated on the sides of each blot.

**9.Blots for Figure 8F (HEC-1A)**

Full-length blots showing protein expression levels in HEC-1A cells under the specified transfection conditions (shNC+vector, sh*ALYREF*+vector, or sh*ALYREF*+*XRCC6*) to evaluate the rescue effects. First row panels: flag-XRCC6 expression levels (left, expected molecular weight: 70 kDa) and its corresponding loading control β-actin (right, 42 kDa). Second row panels: Total β-catenin levels (left, expected molecular weight: 92 kDa) and its corresponding loading control β-actin (right, 42 kDa). Third row panels: Nuclear β-catenin levels (left, expected molecular weight: 92 kDa) and its corresponding nuclear loading control Histone H3 (right, H3, 16 kDa). Fourth row panels: c-Myc levels (left, expected molecular weight: 62 kDa) and its corresponding loading control β-actin (right, 42 kDa).Protein molecular weight markers (kDa) are indicated on the sides of each blot.

**10.Blots for Figure S8F (Ishikawa)**

Full-length blots showing protein expression levels in Ishikawa cells under the specified transfection conditions (shNC+vector, sh*ALYREF*+vector, or sh*ALYREF*+*XRCC6*) to evaluate the rescue effects. First row panels: flag-XRCC6 expression levels (left, expected molecular weight: 70 kDa) and its corresponding loading control β-actin (right, 42 kDa). Second row panels: Total β-catenin levels (left, expected molecular weight: 92 kDa) and its corresponding loading control β-actin (right, 42 kDa). Third row panels: Nuclear β-catenin levels (left, expected molecular weight: 92 kDa) and its corresponding nuclear loading control Histone H3 (right, H3, 16 kDa). Fourth row panels: c-Myc levels (left, expected molecular weight: 62 kDa) and its corresponding loading control β-actin (right, 42 kDa).Protein molecular weight markers (kDa) are indicated on the sides of each blot.
